# Recycling of Undigested Proteins Provided by the Host to the Large Intestine Microbiota: Implication for Intestinal Bacterial Anabolism, Growth, and Physiology

**DOI:** 10.3390/microorganisms13122690

**Published:** 2025-11-25

**Authors:** François Blachier, Xiangfeng Kong

**Affiliations:** 1 UMR PNCA, Université Paris-Saclay, AgroParisTech, INRAe, 91120 Palaiseau, France; 2 Institute of Subtropical Agriculture, Chinese Academy of Sciences, Changsha 410125, China; nnkxf@isa.ac.cn

**Keywords:** undigested proteins, large intestine microbiota, amino acid-derived bacterial metabolites, bacterial metabolism, bacterial physiology, bacterial communication

## Abstract

Although the digestion of dietary and endogenous proteins by the exocrine pancreatic proteases and peptidases in the small intestine luminal fluid is highly efficient for most proteins, it has been roughly approximated that between 3 and 11 g of alimentary proteins and peptides are moving from the small intestine to the large intestine in humans. Here, this nitrogenous material is degraded by the bacterial protease and peptidase activities, releasing amino acids. These amino acids are utilized by the abundant population of bacteria, notably amino acids that the bacteria are unable to synthesize, and which can thus be considered as indispensable for these microorganisms. The anabolism of amino acids by colonic bacteria is related to the synthesis of proteins while some specific amino acids are used for the synthesis of the purine and pyrimidine rings in DNA and RNA. Catabolism of specific amino acids allows for ATP synthesis and results in the production of metabolites with documented roles in the metabolism and physiology of commensal and pathogenic microorganisms among the intestinal microbiota. In the present narrative review, we examine the recycling of the undigested host’s proteins by large intestine bacteria and the metabolism of released amino acids. In addition, we describe how these metabolic pathways are involved in bacterial growth and communication, as well as in bacterial physiology in terms of virulence, resistance to detrimental environmental conditions, and capacity to form biofilms.

## 1. Introduction

The intestine of mammals is well known to lodge a complex mixture of microorganisms, including bacteria, which have been the objects of a vast majority of dedicated studies [1]. These microorganisms, which include also archaea, viruses, and fungi, form the intestinal microbiota [2,3,4,5,6,7], with protozoans forming another component of the gut ecosystem [8].

In healthy individuals, the relatively rapid transit of the luminal content through the small intestine is not compatible with the development of a large concentration of bacteria in the proximal segments of the small intestine. In sharp contrast, the concentration of bacteria greatly increases in the ileum and in the large intestine (cecum, colon, and rectum) [9]. Indeed, in the human colon, the concentration of bacteria represents as much as 10^9^–10^12^ colony-forming units (CFUs) per g of content. This explains why bacteria in human feces represent more than 50% of the total solid part [10].

The spectacular increase in the number of bacteria in the large intestine luminal fluid is notably related to the considerable slowdown of intestinal content transit in the large intestine. This allows for intense utilization by bacteria of the available substrates provided by the host. The transit time in the human colon is rather variable, averaging approximately 60 h [11,12]. In healthy adults, the luminal contents of the distal parts of the intestinal tract are distinguished by much lower values of oxygen tension. Such a condition notably promotes the development of large communities of predominant obligate anaerobes [13]. These bacteria coexist with facultative anaerobes [14].

The substrates available for the growth of bacteria within the colonic fluid are mainly undigested compounds which are transferred through the ileocecal junction from the ileum to the colon [15]. Within the human large intestine, the cecum and the proximal (ascending) colon receive a fluid which is relatively rich in undigested substrates compared to the fluid in the transverse and distal (descending) colon. Regarding the protein part of the undigested substances moving from the ileum to the large intestine, the metabolism of these compounds (referred as putrefaction) in the different parts of the large intestine is poorly documented. However, experimental arguments suggest that protein putrefaction probably occurs mainly within the distal parts of the colon [16,17]. Protein putrefaction is influenced by numerous parameters, including substrate availability, transit time, pH, and osmolarity. The ratio of available carbohydrates/proteins represents an important determinant for relative substrate utilization by gut microbiota [18]; and in humans, the higher the availability of complex carbohydrates (for a given quantity of proteins), the lower microbiota use protein for metabolism [19,20].

When fermentable indigestible carbohydrates are supplied in high amounts to the intestinal microbiota, nitrogenous substrates are used moderately and mainly for some aspects of bacterial anabolism. Conversely, when other sources of energy are scarce, proteins are more intensively degraded and in more diverse metabolic pathways. Longer transit time and more alkaline pH are notably associated with higher levels of protein putrefaction [21,22]. Thus, the carbohydrate/protein ratio influences the overall metabolic activity of the intestinal bacteria, as well as the amounts of amino acids metabolized in the different metabolic pathways [23].

In this narrative review, we present the origin of proteins available for the large intestine bacterial metabolism. Then, the different metabolic pathways responsible for amino acid metabolism in intestinal bacteria are described, notably those involved in the production of bioactive metabolites.

## 2. Origin of Proteins Available for the Large Intestine Microbiota

In the human large intestine luminal fluid, amino acids in their free forms usually do not represent the major nitrogenous sources directly available for microbiota, since amino acid absorption by the enterocytes of the small intestine represents a very highly efficient process [24,25]. Indeed, in the large intestine, amino acids originate mainly from proteins that have not been fully digested in the small intestinal content [26]. Dietary protein digestion is overall an efficacious process, since digestibility in the small intestine is for most dietary proteins equal to or higher than 90% [27,28]. However, some dietary proteins are more resistant to digestion by the catalytic activities of exocrine pancreas proteases. Gluten, for instance, is relatively resistant to digestion [29]. It has been approximated from clinical studies performed in the last decades that between 1.5 and 5 g of nitrogen are transferred every day from the small intestine to the large intestine [30,31,32,33]. The nitrogenous material found in the distal small intestine does not only originate from diet, but, for a major part of it, from endogenous sources. Indeed, roughly 40% of the nitrogenous material transferred to the large intestine originate from alimentation, while the remaining 60% originate from the endogenous compartment [25]. Such nitrogenous material is constituted mainly by proteins and peptides, but also by minor compounds, such as amino acids (approximately 10%), and by small amounts of urea and ammonia [33].

By using the classical conversion factor between nitrogen and proteins, equal to 6.25, by estimating, as explained above, that 40% of the nitrogenous material transferred to the large intestine originates from undigested dietary proteins, and by considering that 90% of the nitrogenous material is made by proteins and peptides, it can be approximated that between 3 and 11 g of alimentary proteins and peptides escape digestion in the small intestine. If we consider the mean protein consumption in the Western world (averaging 85 g/day in adults [34,35]) and the mean dietary protein digestibility in the small intestine (average value 90%), the estimation calculated above is coherent. Indeed, from these last data, we can calculate that an average value of 8.5 g of alimentary proteins (thus 10% of ingested proteins) would be recovered within the large intestine. This value is situated within the 3–11 g range as calculated above [36]. This range can be considered as a rough estimation of the nitrogenous material transferred from the small intestine to the large intestine in humans.

## 3. Metabolism of Proteins by the Large Intestine Bacteria

Proteins are degraded into peptides and amino acids by the large intestine bacteria, and these compounds are taken up by bacteria for further metabolism. The bacterial species living within the human large intestine are, for the most part, unable to synthesize all 22 amino acids required for protein synthesis and utilization in other metabolic pathways. These amino acids can be defined as “bacterially indispensable” for the bacteria concerned.

### 3.1. Degradation of Proteins by the Bacterial Proteases and Peptidases, and Transport of Peptides and Amino Acids in Bacteria

In the large intestine, the numerous bacterial protease and peptidase activities release amino acids from luminal proteins [37]. Bacteria are equipped with a highly diverse set of proteases present in many common gut bacterial species, such as *Clostridium* spp., *Bacteroides* spp., *Lactobacillus* spp., and many others. In fact, hundreds of different proteases have been identified in these bacterial species [38]. Some bacteria, such as lactic acid bacteria, have developed proteolytic systems which compensate for their limited or absent capacities to synthesize several amino acids [39]. Proteolytic systems in lactic acid bacteria gather both extracellular and membrane-bound proteases (notably PrtP and CEP) that convert proteins into oligopeptides. These processes are followed by importation into the bacterial cells via peptide transporters such as Opp, Dpp, and Dtp for oligopeptides, dipeptides, and di- and tripeptides, respectively. Finally, numerous intracellular peptidases in bacteria degrade the oligopeptides into shorter oligopeptides and amino acids [40].

Amino acids and their derivatives can also be both imported and exported from the bacterial cells via transmembrane proteins, including ATP-dependent ABC transporters, different families of channel proteins, and secondary carriers relying on the proton motive force, ionic sodium motive force, and solute–solute exchange [41,42,43]. These amino acid and oligopeptide transporters have been studied in *Escherichia coli* [44]. The uptake of amino acids by large intestine bacteria requires a significant fraction of the available metabolic energy and, as shown in lactic acid bacteria, the metabolic energy cost of amino acid uptake can be significantly reduced by the accumulation of oligopeptides instead of amino acids. Such uptake is followed by the efflux of amino acids which have accumulated in excess in bacteria compared to metabolic needs, with such efflux allowing for the establishment of a proton motive force [45]. Although the relative parts played by oligopeptide and amino acid uptake in the overall accumulation of amino acids within intestinal bacteria are not known, oligopeptide uptake appears significant in bacteria such as lactic acid bacteria. The export of amino acids from intestinal bacterial cells into the luminal fluid may represent a way to supply amino acids to other bacterial species. Such export likely represents a process of metabolic cooperation between different bacterial species. The ways by which colonic bacteria cooperate or compete for available amino acids likely depend on the overall environmental context (abundance or shortage of substrates) [1].

The metabolic fate of amino acids present in free form in large intestine luminal fluid has been studied. Although some measurable transfer of amino acids across the rodent colonic mucosa has been measured in vivo [46], amino acids, in sharp contrast with the situation prevailing in the small intestine, are apparently not absorbed to any significant extent by the mammalian colonic epithelium [47,48,49]. Thus, amino acids entering the large intestine are almost lost for utilization by the host for protein synthesis and for utilization in other metabolic pathways [50].

However, some experimental arguments suggest that some amino acid absorption through the mammalian large intestine epithelium cannot be totally excluded [51]. Firstly, in the pig model, infusion of proteins or amino acids into the large intestine lumen indicate whole-body N balance improvement, which may indirectly suggest some amino acid absorption [52]. Furthermore, some absorption of amino acids from microbial origin through the pig colon has been suggested based on the appearance of ^15^N-labeled amino acids in the venous blood after infusion of ^15^N-labeled bacteria into the cecum [53]. In addition, some biochemical data indicate the presence of ATBo+ neutral and cationic amino acid transporters in the colon [54]. These transporters are localized at the apical pole of colonocytes [55]. Another neutral and cationic amino acid transporter, namely, the Bo+ system, is expressed on the apical surface of colonic absorptive cells [56]. The hypothesis that some amino acids available within large intestine luminal fluid can be used for local protein synthesis within colonic epithelial cells is a possibility that has been little evaluated [57]. Some amino acid absorption by the colon epithelium has been measured in a pig model during the neonatal period [58,59], suggesting a transient colonic absorption of amino acids in this experimental model.

Finally, from the available data, it appears that amino acids within the colon luminal fluid in adults are available, presumably, for the most part, for the metabolic activity of the microbiota in relationship with their growth and physiology, as will be detailed in the following sections.

### 3.2. The Indispensable Amino Acids for Intestinal Bacterial Species

The definition of indispensable amino acids in mammals (including humans) is related to the fact that, among the amino acids required for metabolism and physiological functions in the tissues and organs, nine of them (namely, isoleucine, leucine, valine, lysine, methionine, phenylalanine, threonine, tryptophan, and histidine) must be provided by the diet to meet the requirements [60,61]. In other words, indispensable amino acids are those which the body cannot synthesize, or cannot synthesize to any significant extent, when compared to the metabolic and physiological needs.

By analogy with the definition of indispensable amino acids in mammals, it is tempting to propose the use of the same terminology with some nuances for the intestinal bacteria. Indeed, not all bacteria within the large intestine are able to synthesize the 20 usual amino acids and the 2 unusual amino acids (namely, selenocysteine and pyrrolysine) they need for protein synthesis and for utilization in other metabolic pathways [23]. The amino acids which cannot be synthesized by the intestinal bacteria (or cannot be synthesized in a significant amount to cover the requirement) must be supplied from the luminal fluid and imported within the bacterial cells.

By deduction, the non-indispensable (or dispensable) amino acids for the large intestine bacteria are those which can be synthesized in a sufficient amount to cover their metabolic and physiological requirements. The studies of amino acid biosynthesis in intestinal bacteria have been historically focused on a few bacterial species, notably including *Escherichia coli*, as well as enterohemorrhagic *E. coli* (EHEC), which is responsible for severe colonic infection [62], *Salmonella typhurium*, a pathogen known to provoke diarrhea and inflammation in ileum and colon [63], and *Bacillus subtilis*, which is present in the human gut and which has shown some protective effects in *Citrobacter rodentium*-associated colitis [64]. Obviously, the available metabolic data related to these few bacterial species cannot be considered as representative of amino acid anabolism for all the bacterial species present within the large intestine. With this reservation of the heterogeneity of bacterial metabolic capacities in mind, most of the operative metabolic pathways involved in amino acid metabolism appear conserved across the bacterial lineages studied. The description of the metabolic pathways involved in the biosynthesis of the 22 amino acids in bacteria is outside the scope of this review but can be found in recent reviews dedicated to this topic [23,38].

The diversity of the metabolic capacities for the different bacteria living in the gut can be illustrated by some typical examples. *Clostridium perfringens*, a common human enteropathogen [65], lacks for instance one or several genes involved in threonine, serine, glutamate, arginine, histidine, lysine, methionine, aromatic, and branched-chain amino acid biosynthesis [66]. This pathogen thus depends on the presence of these amino acids in the surrounding media. Therefore, these amino acids can be considered as indispensable for *Clostridium perfringens*.

Regarding *Lactobacillus johnsonii*, a human gut commensal [67], this bacterium is unable to synthesize almost all amino acids due to the lack of complete biosynthetic pathways, thus exhibiting dependence on most amino acids either in free form or supplied in peptidic forms in media [68]. Other bacteria, like *Campylobacter jejuni*, a foodborne pathogen which causes inflammation and enteritis in humans [69], and *Enterococcus faecalis* strains, which are resident intestinal bacteria associated with invasive infections and inflammatory bowel diseases [70], do not contain the whole biosynthetic pathways for some amino acids [71].

Here, it is important to recall that the sole presence of genes involved in amino acid synthesis within a bacterial genome is not sufficient to establish the functionality of the corresponding pathways [38]. For instance, the genes for all the 20 common amino acids have been identified in the bacterium *Lactococcus lactis*, a noninvasive and nonpathogenic organism [72]; and despite this result, 6 amino acids, namely, glutamate, methionine, isoleucine, valine, leucine, and histidine, need to be present in media to allow for bacterial growth. This is because, due to point mutations, genes coding for several enzymes involved in the biosynthetic pathways of these amino acids are nonfunctional [73,74]. The same situation is found for *Staphylococcus aureus*, a common colonizer of the human gut [75]. Indeed, this bacterium exhibits the absolute requirement for cysteine, proline, arginine, valine, and leucine, despite the presence of the complete sets of genes corresponding to the different biochemical pathways responsible for the biosynthesis of these amino acids [76].

### 3.3. Utilization of Amino Acids for Synthesis of Macromolecules in Bacteria

**Figure 1:**
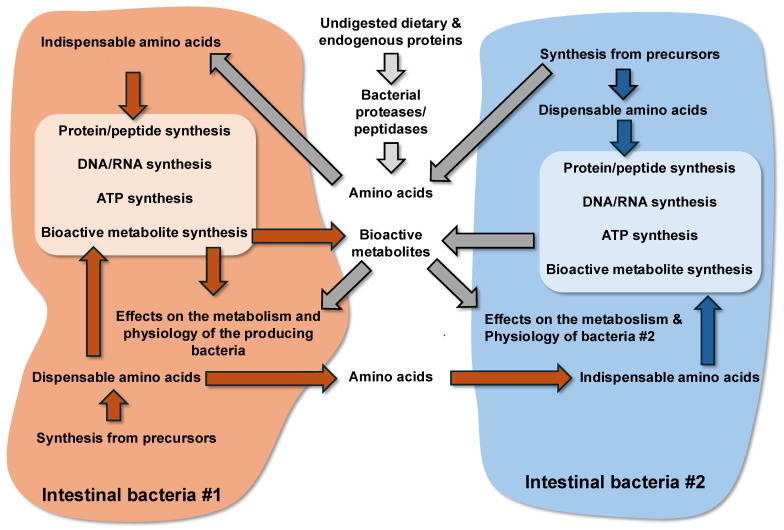
Schematic view of the metabolism of amino acids by the intestinal bacteria.

Amino acids in bacteria are well known to be utilized for the synthesis of macromolecules, including not only proteins but also for RNA and DNA synthesis (the main pathways for the metabolism of amino acids by the intestinal bacteria are summarized in Figure 1). To face a changing environment, bacteria depend on strictly coordinated proteostasis networks that finally control processes such as the rates of protein synthesis and degradation [77]. One critical mechanism that is involved in the response of bacteria to a changing environment (including changes in the nutrient concentrations) is represented by the control of the expression of genes at the level of proteosynthesis. At each of the three major steps of translation, namely, initiation, elongation, and termination, bacterial cells can tune the translation rate and thus the intracellular protein concentrations depending on their environment [78]. Of note, protein synthesis and protein turnover are dependent on post-translational modifications of a group of bacterial proteins [79].

Regarding RNA and DNA synthesis, three amino acids, namely, glycine, aspartate, and glutamine, are used as precursors for the synthesis of the purine and pyrimidine rings. The metabolic pathways involved in pyrimidine biosynthesis from amino acids have been studied in numerous bacterial species, and the results have shown that the synthesis of pyrimidines requires carbamylphosphate as a precursor. In *Escherichia coli*, *Salmonella typhimurium*, and other bacterial species, including *Pseudomonas aeruginosa* (present in the human intestinal tract [80]) and *Proteus mirabilis* (which is associated with inflammation in Crohn’s disease [81]), carbamylphosphate is synthesized from the condensation of glutamine and bicarbonate in an ATP-dependent reaction. Then, in the first step of pyrimidine synthesis, carbamylphosphate and aspartate are converted to carbamylaspartate. This latter compound is then used as precursor for uridine triphosphate and cytidine triphosphate in six and seven steps, respectively [82]. Interestingly, the pool of intracellular nucleotides in bacteria ultimately regulates protein synthesis at various stages of this process [83]. This shows the interrelationships between the different metabolic pathways.

### 3.4. Utilization of Amino Acids for the Synthesis of ATP in Bacteria

Under aerobic conditions, some bacterial species convert, via transamination and deamination reactions, amino acids to their α-ketoacid counterparts. These α-ketoacids can then be oxidized in the citric acid cycle. In addition to these metabolic pathways, some bacteria are equipped with a branched-chain keto acid dehydrogenase complex, whose catalytic activity is associated with ATP synthesis [84]. However, under strict anaerobic conditions, such as those found in the colon, or in the absence of any suitable electron acceptor, strict or facultative anaerobic bacteria, such as Clostridia and Fusobacteria, can utilize amino acids as energy sources. They use amino acids in a wide range of reactions catalyzed by numerous enzymes involved in the reactions of transamination, oxidation, and reduction [85,86]. In the absence of oxygen and/or of other inorganic electron acceptors, the substrates for fermentation reactions are used both as electron donors and as electron acceptors. This results in a modest ATP synthesis yield when compared to the yield measured under aerobic conditions [87]. Many electron donors and acceptors can participate in the different biochemical pathways involved in protein putrefaction. These compounds include α-ketoacids, molecular hydrogen (H_2_), as well as amino acids [88,89]. Bacterial amino acid utilization results in the production of different end products, mainly including short- and branched-chain fatty acids [84].

In addition, bacteria, such as lactic acid bacteria, can use amino acid decarboxylation to generate ATP [90,91]. Amino acid decarboxylation presumably supports ATP production in adverse environmental conditions. For instance, tyrosine decarboxylation in bacterial species equipped with this metabolic pathway results in the production of tyramine and carbon dioxide (CO_2_) and allows the production of ATP by utilizing the proton motive force. This results in both a pH gradient formed during proton consumption in the decarboxylation reaction and in the formation of a membrane potential, which itself apparently results from electrogenic transport of tyrosine in exchange for tyramine [92].

In *Clostridium* species, including *Clostridium difficile*, specific metabolic pathways grouped under the name of Stickland metabolism is central for ATP production. Stickland reactions are known to involve the coupled oxidation and reduction of two amino acids, with one amino acid acting as an electron donor and another amino acid acting as an electron acceptor. These later reactions allow to produce ATP by substrate-level phosphorylation and by the maintenance of the NADH/NAD^+^ pools [93]. Efficient electron donors include amino acids such as isoleucine, alanine, valine, and leucine, while efficient electron acceptors include amino acids such as leucine, proline, and glycine [94]. Ornithine, a non-proteogenic amino acid, can also undergo Stickland metabolism, allowing for the synthesis of acetyl CoA, ammonia, and alanine, or can be alternatively converted to proline [95]. By doing so, ornithine participates in the production of ATP and in the synthesis of specific amino acids in the bacterial species equipped with these metabolic capacities. Stickland amino acid reactions allow for the provision of metabolic resources to support bacterial growth in situations when other substrates, such as carbohydrates, are in short supply [96].

Of note, amino acid utilization for ATP production may be operative in a preferential manner when compared to the utilization of other ATP-producing substrates. For instance, *Clostridium sticklandii*, a nonpathogenic proteolytic clostridium, which is known to be dependent on amino acids for growth, is considered as a “specialist” for amino acid catabolism. This bacterium has been studied in detail for its metabolic capacity to degrade amino acids, thus allowing ATP production [97]. *Clostridium sticklandii* is characterized by numerous transporters for oligopeptides and amino acids, and with a battery of proteolytic enzymatic activities at the origin of these compounds. This bacterium preferentially utilizes amino acids such as serine, glycine, arginine, threonine, and cysteine for ATP synthesis.

Interestingly, several amino acids are used by some bacterial species in specific conditions of growth. For instance, lysine degradation represents a major source of ATP for *Clostridium sticklandii* only in stationary growth phase.

### 3.5. Amino Acid Utilization for the Synthesis of Bioactive Metabolites in Bacteria and Effects of These Compounds on Bacterial Growth and Physiology

Numerous amino acid-derived metabolites synthesized as intermediary or end products by different intestinal commensal and pathogenic bacteria have been shown to be active on their own metabolism and physiology, with effects on their virulence and growth capacities [98]. In addition, from recent studies, the emerging roles of several amino acid-derived metabolites released in the surrounding media by some bacterial species have been presumed to act on other bacterial species. This suggests that the amino acid bacterial metabolism may be involved in the regulation of bacterial physiology, and likely as a means of communication between bacteria in different ecosystems. Studies on communication between bacteria generally refer to events involved in the capacity of bacteria to collectively modify their physiology in a changing environment. Such events include both ligand/receptor binding and uptake through bacterial membrane.

*Polyamines derived from specific amino acids facilitate bacterial growth, modulate bacterial virulence, regulate biofilm formation, and promote bacterial resistance to acidity*

**Figure 2:**
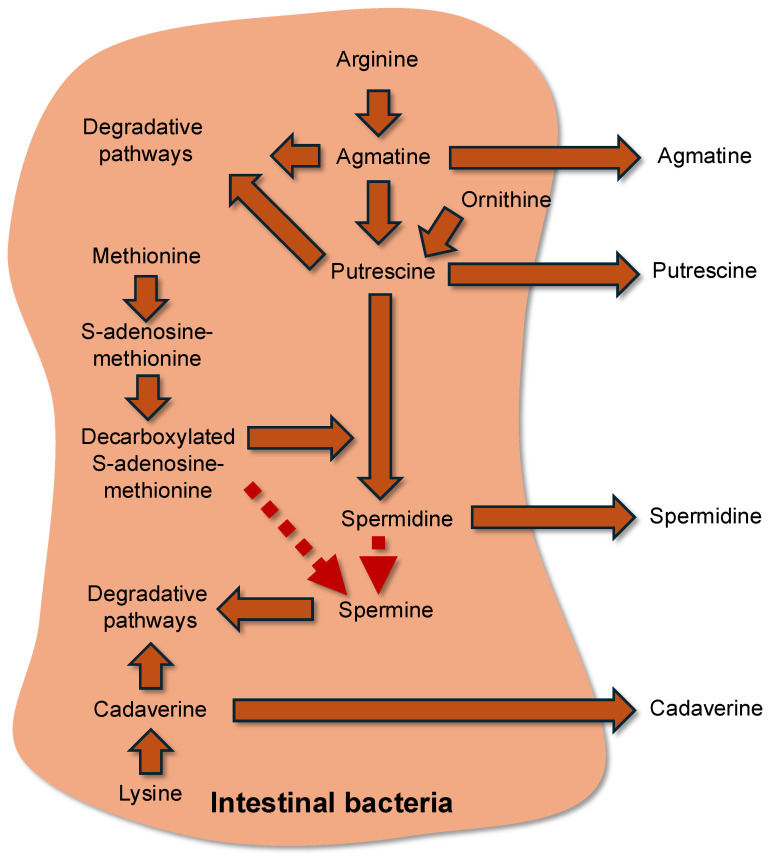
Schematic view of the metabolism of polyamines by intestinal bacteria.

Polyamines represent a family of small aliphatic amines that are produced by bacteria from specific amino acids. Putrescine, spermidine, agmatine, and cadaverine are the main polyamines synthesized by bacteria. However, numerous polyamine derivatives can be synthesized by bacteria from these polyamines, and for several of them, by pathways that are not operative in eukaryotic cells [99,100]. Putrescine and spermidine are commonly found at relatively high concentrations in bacteria, while agmatine and cadaverine are found at lower concentrations, and spermine is rarely detected in most bacterial species [101]. Polyamines are found in the intestinal contents at concentrations from micro- to millimolar concentrations [1]. A part of polyamines within bacteria is associated with RNA, and such association corresponds to the effects of polyamines on transcriptional and translational processes [102,103,104]. The amino acids ornithine, arginine, and methionine are the precursors for the synthesis of putrescine and spermidine, while arginine is the precursor for agmatine synthesis. Regarding cadaverine, this polyamine is produced from lysine [101]. The methyl donor S-adenosylmethionine is required for the conversion of putrescine to spermidine, and for the conversion of spermidine to spermine, although this latter reaction is rarely detected in common bacteria (Figure 2).

However, not all bacteria are equipped with metabolic machinery allowing for the biosynthesis of all polyamines from amino acid precursors. This indicates that some bacterial species are dependent on the import of polyamines present in media for specific actions, as detailed in the following paragraph [105]. These polyamines likely originate from the polyamines synthesized and released by other members of the bacterial community. This is coherent with the identification of several systems of polyamine uptake and release, which have been characterized in numerous bacterial species, including those inhabiting the large intestine. For instance, agmatine–putrescine antiporters have been characterized in *Enterococcus faecalis* [106]. Polyamine degradation pathways represent another important component in fixing the polyamine concentrations within the bacteria intracellular medium [107]. Thus, polyamine concentrations in bacteria are the net result of complex processes such as endogenous synthesis, import, export, and degradation.

Polyamines participate in critical physiological functions in intestinal bacteria. The effects of polyamines on bacterial growth are rather heterogeneous depending on the species studied. For instance, *Escherichia coli* rendered deficient for polyamine synthesis can still grow, albeit at a reduced rate, when compared with the wild-type counterpart. These results show that polyamines are not strictly required for the growth of this bacterium but are required for optimal growth [108]. Likewise, in the foodborne pathogen *Salmonella enterica*, polyamine depletion reduces but does not suppress bacterial cell growth [99]. In contrast, polyamine biosynthesis is essential for the growth of the pathogen *Pseudomonas aeruginosa* and of the foodborne pathogen *Campylobacter jejuni* [109,110].

Regarding the implication of polyamines in bacterial metabolism, spermidine modulates bacterial toxin production. Indeed, this last compound increases for instance the production of colibactin [111]. Regarding cadaverine, this polyamine can attenuate virulence and reduce enterotoxin activity in *Shigella spp*. In addition, putrescine, cadaverine, spermidine, and spermine have been shown to modify the virulence of *Vibrio cholerae* [112].

Polyamines can be incorporated into bioactive compounds, such as bacterial siderophores. These compounds are secreted by bacteria and then scavenge iron in the extracellular medium. Iron is then supplied to bacteria via specific receptors [113]. Spermidine and cadaverine can react with bacterial cell wall compounds, such as peptidoglycans, and this process presumably increases bacterial cell wall rigidity [114]. Cadaverine has been demonstrated to provide a mechanism to *Escherichia coli* and the foodborne pathogen *Vibrio parahaemolyticus* involved in resistance to increased acidity [115,116].

Lastly, polyamines are involved in the regulation of biofilm formation. Briefly, biofilms can be viewed as a mixture of high-density bacterial (and archaeal) communities contained within a self-producing protective matrix made of polysaccharides, proteins, nucleic acids, and lipids [117,118]. Such structures represent an important element for the modulation of bacterial growth in an evolving environment, like the one which is found in the large intestine [119]. Biofilms are found in the intestine where elements of the intestinal microbiota contained within these structures are in proximity with the intestinal mucosal surface [120]. Research on biofilms is notably motivated by the fact that the intestinal pathogens in biofilms are generally much more resistant to treatments with antimicrobial agents [121] than pathogens not present is these structures. Biofilm development and quorum sensing have been shown to be interconnected [122,123]. In a few words, quorum sensing is related to the capacity of bacteria to collectively modify their physiology in response to changes in the cell density and species composition within the local environment [124]. The implication of different polyamines in the regulation of biofilm formation, either positive or negative, has been recently documented. The polyamine agmatine is involved in biofilm formation in *Bacillus subtilis* [125]. Another polyamine, namely, the uncommon polyamine norspermidine, reinforces biofilm formation due to increased cyclic di-GMP synthesis. This last compound can by itself activate the expression of genes involved in the formation of biofilm components in *Vibrio cholerae* [126]. In an intriguing and interesting way, norspermidine appears to act on *Vibrio cholerae* via a norspermidine sensor [127]. An additional study has confirmed that norspermidine biosynthesis is required for biofilm formation by *Vibrio cholerae* [128]. In contrast, spermine inhibits *Vibrio cholerae* biofilm formation [129]. The results presented above indicate that different polyamines can exert opposite effects on the bacterial capacity to form biofilms. The respective roles of the different polyamines (either synthesized endogenously by bacteria or taken up from the extracellular media) for the regulation of biofilm formation by different bacterial species among the intestinal microbiota remain unclear, thus requiring additional experimental works.

*Cysteine-derived hydrogen sulfide increases bacterial respiration and growth, promotes biofilm formation, and modulates the effects of different antibiotics*

Hydrogen sulfide (H_2_S) is produced in bacteria from different S-containing substrates, notably cysteine and inorganic sulfate [130]. Cysteine-degrading bacteria in the gut include *Fusobacterium*, *Clostridium*, *Escherichia coli*, *Salmonella*, *Klebsiella*, *Streptococcus*, and *Enterobacter*. These bacteria convert cysteine to H_2_S through the catalytic activity of cysteine desulfhydrase [131,132,133]. Sulfate-reducing bacteria include the following gut bacteria: *Desulfobrio*, *Desulfobacter*, *Desulfobulbus*, and *Desulfomaculum* [134,135,136]. *Desulfovibrio* represents the dominant genera of sulfate-reducing bacteria in the intestine [137,138,139]. The amount of sulfate in the diet, as well as the capacity of the small intestine to absorb this compound, appear to represent the main parameters which determine the amount of sulfate transferred to the large intestine [140]. In volunteers, the ingestion of sulfate in supplement increased the fecal sulfide production rate [141]. It appears that, overall, current sulfate concentrations in the large intestine are adequate to support the growth of sulfate-reducing bacteria.

H_2_S in the colonic luminal fluid can exist in three forms: the H_2_S gas that is partly dissolved in the aqueous phase and represents a highly diffusible compound, hydrosulfide anions (HS^−^), and sulfide ions (S^2−^). This latter compound is likely present at a negligible level in the colon. Indeed, in the aqueous phase of the colonic fluid, H_2_S dissociates into HS^−^ and S^2−^ and H^+^, with pKa values of 7.04 and 11.96, respectively [142,143]. In healthy subjects, the pH at the colonic mucosal surface ranges between 7.2 and 7.5 in the descending colon and the rectum [119]. Thus, by considering a pH equal to 7.4, approximately one-third of sulfide is in the form of H_2_S at equilibrium, while two-thirds are in the form of hydrosulfide anions. However, if the pH becomes more acidic, the H_2_S/HS^−^ ratio increases in the large intestine. The measurement of H_2_S concentrations measured in the colonic luminal fluid led to rather divergent values according to the techniques used, ranging from high micromolar to low millimolar concentrations [1]. Measurement in the feces obtained from human volunteers indicate that 8% of sulfide is in the unbound form [144]. The identification of the compounds which bind sulfide in the colonic lumen, although obviously not exhaustive, points out several compounds from a dietary origin that are not fully absorbed in the small intestine. These compounds include zinc [145], heme [146], and polyphenols [147].

**Figure 3:**
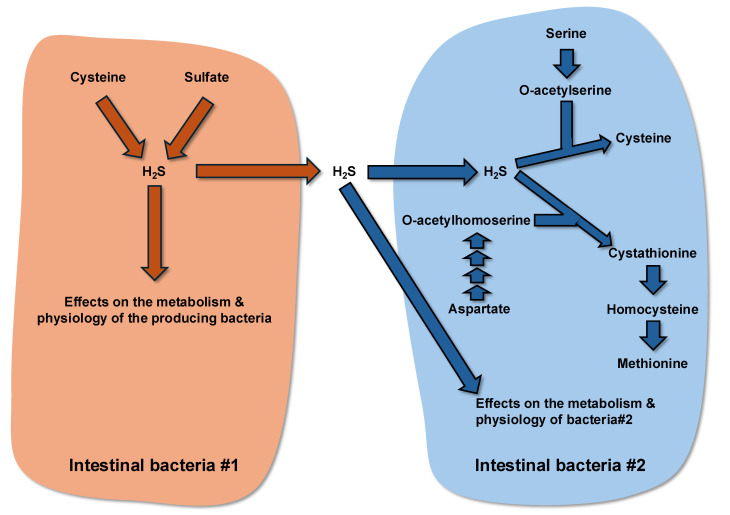
Schematic view of the metabolism of hydrogen sulfide by the intestinal bacteria.

Incidentally, H_2_S is involved as a precursor for the synthesis of cysteine and methionine in bacteria. Such anabolic pathways in gut bacterial species may influence the sulfide concentration within the intestinal content. In bacteria, the synthesis of cysteine can be made from serine in two consecutive steps, with the first step being catalyzed by serine acetyltransferase in the presence of acetyl CoA, and the second step being catalyzed by cysteine synthase with the involvement of H_2_S as co-substrate [148,149]. Some bacteria are not equipped with cysteine synthase. For instance, *Bifidobacterium longum*, which is abundant in the infant and adult intestine [150], lacks the corresponding gene [151]. Regarding methionine synthesis in bacteria, two pathways have been described, with one involving H_2_S. In this pathway, o-acetylhomoserine synthesized from aspartate reacts with H_2_S (or cysteine), allowing cystathionine production. Cystathionine is then converted to homocysteine, which is converted to methionine by the enzyme methionine synthase [152,153] (Figure 3).

At low concentrations, H_2_S stimulates the respiration of the intestinal pathogen *Mycobacterium tuberculosis* and promotes its growth [154]. In contrast, at high concentrations, H_2_S inhibits the terminal oxidase of the respiratory chain of *Escherichia coli* [155]. Terminal oxidase represents one element of the bacterial respiratory chain [156]. However, *Escherichia coli* contains an alternative bd-type oxidase, which is insensitive to the inhibitory effects of H_2_S. Then, the presence of this isoenzyme allows for respiration and the associated growth of this bacterium in a H_2_S-rich environment [157]. In a similar way, cyanide-insensitive oxidase in the intestinal pathogen *Pseudomonas aeruginosa* confers tolerance to H_2_S for its respiration [158]. Thus, it appears that an alternative solution for respiration exists in some intestinal bacteria to cope with an increased sulfide luminal concentration.

H_2_S can also act on some intestinal bacterial species as a protective compound. In the pioneering publication of Bachenheimer and Bennett, the authors presented experimental arguments suggesting that H_2_S produced by *Desulfovibrio desulfuricans* is the diffusible factor responsible for the protection of *Pseudomonas aruginosa* from the toxicity of heavy metals [159]. These results, published more than six decades ago, indicate that a given bacterial species was able to produce a compound active towards another bacterial species. Similarly, H_2_S produced by *Escherichia coli* can contribute to the protection of another bacterial species, namely, *Staphylococcus aureus*, against toxicity by mercury [160]. Since these discoveries, but much more recently, in the context of the study of bacterial antibiotic resistance, H_2_S has emerged as a protective compound for *Pseudomonas aeruginosa* and *Staphylococcus aureus* against the action of different antibiotics [161,162]. Although the precise mechanisms of action involved in the protective action of H_2_S against the effects of antibiotics are not known, several experimental results have shed light on these mechanisms. One mechanism in *Escherichia coli* is the sequestration of Fe^2+^ ions by H_2_S, which counteracts the oxidative stress triggered by some antibiotics [163]. Furthermore, H_2_S is involved in the maintenance of the bacterial redox homeostasis and protects pathogenic *Escherichia coli* strains against the oxidative stress triggered by the antibiotic ampicillin [164].

Cystathionine-Ɣ-lyase has been discovered as the primary enzymatic activity responsible for H_2_S production in *Staphylococcus aureus* and *Pseudomonas aeruginosa*. Interestingly, the inhibition of this activity reinforces antibiotic efficiency against both bacterial species in in vitro and in vivo models of infection [165]. Overall, these results suggest that endogenously formed H_2_S participates in the resistance of some bacteria against the effects of some antibiotics.

However, H_2_S is apparently not a bacterial amino acid-derived metabolite which limits the efficiency of antibiotics against all bacteria present within the intestine. For instance, in *Acinetobacter baumannii*, which incidentally is not a H_2_S producer, H_2_S reinforces the effects of several classes of antibiotics [166]. Thus, the H_2_S-mediated protection (or, conversely, sensitization) to the effects of different antibiotics is dependent on the bacterial species studied and the antibiotics used. Finally, in another context, the implication of H_2_S in the resistance to infection by pathogenic bacteria has been suggested [167]. Indeed, in this last study, the capacity for the endogenous production of sulfide apparently represents one parameter involved in the enhanced capacity of intestinal commensal bacteria to counteract pathogenic infection.

Regarding the establishment and restoration of colonic microbiota biofilms, H_2_S has been shown to intervene in such processes [168]. *Fusobacterium nucleatum* is a H_2_S producer, and the production of this metabolite in this bacterial species modulates the virulence and susceptibility to antibiotics [169]. The scavenging of H_2_S in several bacterial species (including *Escherichia coli*) which produce this compound potentiates both the bactericidal effects of several active compounds and disrupts the formation of the bacterial biofilms, thus indicating that the endogenous synthesis of H_2_S by these species represents one component involved in biofilm formation, and presumably the associated resistance to bactericidal agents [170].

*Nitric oxide derived from arginine interferes with bacterial energy metabolism and growth, and can increase the dispersal of biofilms*

Nitric oxide (NO) is produced from the amino acid arginine by nitric oxide synthases found in numerous bacteria [171], including several bacteria found in the intestine, such as *Bacillus subtilis* and *Lactobacillus fermentum* [172,173]. Nitric oxide, as documented for hydrogen sulfide, is a highly diffusible gaseous compound. Incidentally, it is worth noting that the nitric oxide synthase pathway is not the exclusive means for nitric oxide production in intestinal bacteria. In fact, intestinal bacteria such as *Campylobacter jejuni* and *Pseudomonas aeruginosa* can produce NO in the process of nitrite (NO_2_^−^) reduction catalyzed by nitrite reductase [174,175,176]. NO can also be formed by ammonia oxidizing bacteria, such as bacteria belonging to the *Nitrospira* genus [177], with some of them being found in the human intestine [178]. The concentration of NO in the gaseous phase of the colon has been measured in volunteers and average approximately 20 p.p.m. [179].

NO has been experimentally shown to interfere with bacterial growth and is involved in the biosynthesis of several bacterial compounds. Indeed, NO exerts bacteriostatic effects against several intestinal bacteria, such as *Salmonella enterica* [180]. NO appears to exert its bacteriostatic action at least partially by inhibiting enzymatic activities related to bacterial energy metabolism [181]. Apart from these effects on bacterial growth, NO has been involved in the synthesis of some bacterial metabolites. For instance, NO is used as a building block for the synthesis of the 1,2,3 triazole moiety of 8-azoguanine [182], a member of the purine and pyrimidine antimetabolites, but it remains unknown if 8-azoguanine is effectively produced by any bacterial species belonging to intestinal microbiota. NO intervenes in nitration reactions within bacteria [183]. For instance, the bacterial compound rufomycin is produced by bacteria in a NO-dependent nitration step. Rufomycin is known to be bioactive and to target proteolysis in *Mycobacterium tuberculosis* [184].

Lastly, NO allows for the rapid dispersal of the biofilm macrostructure by mechanisms involving NO-sensory proteins in many pathogenic bacteria, including the intestinal pathogen *Vibrio cholerae* [185]. Accordingly, several NO donors with different chemical structures have been tested recently on different bacterial species, and the results of these experiments show the antimicrobial and antibiofilm effects of these compounds, notably on *Fusobacterium nucleatum* [186]. In the bacterial species *Vibrio parahaemolyticus*, which may act as an intestinal pathogen, NO increases the transcription of the quorum sensing regulatory gene *opaR* [187]. Although NO emerges as a gasotransmitter active in the regulation of biofilm formation, further work is required to test if this compound produced endogenously by intestinal bacteria reaches concentrations within the luminal fluid that are compatible with this effect.

*Indole derived from tryptophan diminishes bacterial growth and virulence*

Numerous Gram-positive and Gram-negative bacterial species can produce indole from the precursor tryptophan, notably the intestinal bacterial species *Escherichia coli*, *Proteus vulgaris*, *Clostridium* spp., and *Bacteroides* spp. [188,189,190]. The indole concentration has been measured in the feces of volunteers, thus reflecting the concentration of indole in the most distal segment of the large intestine, and has been found to range from 0.3 to 6.6 millimolar [1]. Indole diminishes the capacities of bacterial cells for motility and aggregation in *Listeria monocytogenes*, a bacterial species occasionally found within the intestine [191]. Indole is also able to diminish the virulence of intestinal bacterial species, such as *Pseudomonas aeruginosa* and *Salmonella enterica* [192,193].

Importantly, indole is also active in diminishing the virulence and growth of the fungal species *Candida albicans* [194], a microorganism commonly found in the intestine [195]. This discovery reveals communication between intestinal bacteria and fungi via the production of a specific amino acid-derived bacterial metabolite. Lactic acid bacteria are also sensitive to the effect of indole, since this amino acid-derived metabolite shows a bacteriostatic effect on these bacteria [196]. Lastly, indole affects toxin production by *Klebsiella oxytoca* [197], a normal resident in the intestine which may become pathogenic according to the environmental context [198].

*Tyrosine-derived p-cresol produced by Clostridium difficile gives competitive advantage to this bacterium over other bacteria*

The bacterial metabolite *p*-cresol (4-methylphenol), which originates from the amino acid tyrosine, can be produced by anaerobic bacteria, notably the ones found in the large intestine luminal fluid [199]. Among these numerous bacteria, specific families of bacteria, like *Fusobacteriaceae*, *Enterobacteriaceae*, *Clostridium*, and *Coriobacteriaceae*, are active *p*-cresol producers [200,201]. The *p*-cresol concentrations measured in the human colonic contents remain in the low millimolar range [1]. Of major interest, the capacity of *Clostridium difficile* to produce *p*-cresol is one important element which gives this bacterial species a competitive advantage over other gut bacteria, such as *Escherichia coli*, *Klebsiella oxytoca*, and *Bacteroides thetaiotaomicron* [202]. *Clostridium difficile* is well known to represent a major cause of intestinal infection and diarrhea in patients following treatment with antibiotics [203]. Using a mouse model of *Clostridium difficile* infection, it has been observed that excessive *p*-cresol production affects gut microbiota diversity. Also, by removing the capacity of *Clostridium difficile* to produce *p*-cresol, this bacterium was less able to recolonize the intestine after an initial episode of infection [202]. As expected, *Clostridium difficile* can tolerate *p*-cresol concentrations as high as 10 millimolar [204,205].

*Skatole derived from tyrosine inhibits biofilm formation by enterohemorrhagic Escherichia coli*

Skatole (3-methylindole) is a metabolite produced by specific bacteria from tryptophan [206,207]. For instance, *Lactobacillus*, *Clostridium*, and *Bacteroides* are known as skatole producers [208]. Fecal skatole concentrations in healthy individuals are usually relatively low, averaging approximately 40 micromolars [1]. Skatole displays an efficient capacity to inhibit biofilm formation by the enterohemorrhagic *Escherichia coli* [209].

*Glycine-derived betaine protects bacteria against osmotic stress*

Although glycine betaine metabolism has been principally studied in bacteria not found in the intestine, these studies indicate that this compound is derived from extracellular sources and/or from endogenous synthesis from glycine [210,211]. Glycine betaine can protect bacteria against osmotic stress [212]. Notably, glycine betaine is an osmoprotectant for the intestinal bacteria *Pseudomonas aeruginosa* [213]. Such a capacity may be relevant in the case of increased luminal osmolarity within the colonic fluid [119], but this aspect needs to be further documented in future works.

*Amino acid-derived glutathione is active against oxidative stress*

Glutathione is synthesized from the three precursors glutamate, cysteine, and glycine by bacteria such as *Listeria monocytogenes*. Glutathione plays a key role in maintaining the proper oxidation state of protein thiols in bacteria, notably in the context of oxidative stress [214,215].

*Histidine-derived histamine helps bacteria to survive in acidic media*

Histamine, mainly known as a biogenic amine involved in allergic diseases, has been demonstrated to act on some bacteria. The production of histamine from the decarboxylation of the amino acid histidine has been demonstrated in numerous Gram-positive and Gram-negative bacterial strains [216]. These bacteria notably include bacteria belonging to the intestinal microbiota, such as *Pediococcus parvulus*, *Morganella morganii*, *Klebsiella pneumoniae*, *Enterobacter* spp., *Citrobacter freundii*, and *Hafnia alvei* [217]. Recent studies show that histamine can improve the survival of bacteria in acidic conditions. The generation of the proton motive force by histidine decarboxylation has been demonstrated in *Lactobacillus buchneri* [218]. Furthermore, it has been elegantly shown that, by expressing the functional histidine decarboxylase pathway in *Lactococcus lactis*, these bacteria survive longer in an acidic medium (pH 3.0) than their control cell counterparts [219]. However, since the pH in the human large intestine is usually only slightly acidic or near neutrality, ranging from 5.7 to 6.8 in the cecum, while ranging from 6.1 to 7.5 in the descending colon and rectum [220], it remains to be demonstrated if such histamine-dependent capacity of bacteria to survive in an acidic medium is relevant for intestinal bacteria.

*Amino acid-derived dopamine, serotonin, noradrenaline, and gamma-amino butyric acid are involved in the regulation of bacterial growth and virulence, and in the tolerance to increased acidity*

Dopamine, primarily known as a neurotransmitter in animals, has been shown to be active on some bacteria. Dopamine is produced by many bacterial species present in the intestine, such as *Bacillus subtilis*, *Escherichia coli*, *Staphylococcus aureus*, *Proteus vulgaris*, and *Klebsiella pneumoniae* [221]. Bacterial tyrosinases are enzymatic activities which catalyze in bacteria the conversion of tyrosine to dihydroxyphenylalanine (DOPA), the direct precursor of dopamine [222]. Dopamine concentration has been measured in the mouse large intestine luminal content, averaging approximately one micromolar [223]. To the best of our knowledge, the concentration of dopamine in the human colonic fluid has not been determined. Dopamine accelerates the growth of *Pseudomonas aeruginosa* and *Klebsiella pneumoniae* [224]. Furthermore, dopamine is a siderophore-like iron chelator that is involved in the optimal growth of *Salmonella enterica* [225].

Serotonin (5-hydroxytryptamine), a compound which is well known to be a neurotransmitter in animals, is produced from the amino acid tryptophan in many intestinal bacterial species, among which *Propionibacterium*, *Lactobacillus*, *Lactococcus*, *Bifidobacterium*, *Streptococcus*, *Bacteroides*, and *Escherichia coli* [217]. Serotonin appears to regulate either positively or negatively, depending on the bacterial species considered, the virulence of different bacteria. For instance, serotonin positively affects the virulence of *Pseudomonas aeruginosa* in both in vitro experiments and in vivo tests performed in a model of rodent infection [226]. In this latter study, serotonin was found to participate in the regulation of bacterial quorum sensing.

Noradrenaline (norepinephrine) is produced from the amino acid tyrosine by several intestinal bacteria, including *Bacillus subtilis*, *Escherichia coli*, and *Proteus vulgaris* [217]. This compound, known as neurotransmitter in animals, affects bacterial growth either through growth stimulation or inhibition, depending on the bacterial species studied and on the experimental context. Noradrenaline is notably active on the growth of the following intestinal anaerobic bacteria: *Klebsiella pneumoniae*, *Fusobacterium nucleatum*, *Pseudomonas aeruginosa*, *Enterobacter clocae*, *Shigella sonnei*, and *Staphylococcus aureus*. In addition to its effect on bacterial growth, noradrenaline increases the virulence of several anaerobic bacteria, including *Clostridium perfringens* [227,228,229].

Gaba-amino butyric acid (known as GABA) is produced from the amino acid glutamate by several bacterial species present in the intestinal luminal fluid, such as *Lactobacillus* and *Bifidobacterium* [230,231,232]. GABA, known as the main inhibitory neurotransmitter in the mammalian brain, is involved in the tolerance of bacteria, such as *Bacteroides* spp., to acidic media through the maintenance of the intracellular pH [233,234].

*4-hydroxyphenylacetate derived from tyrosine reduces bacterial growth and virulence*

The bacterial metabolite 4-hydroxyphenylacetate represents an intermediary metabolite produced from the amino acid tyrosine by intestinal bacterial species during phenol and *p*-cresol synthesis [200]. The transport of 4-hydroxyphenylacetate from the extracellular media is also possible and has been studied in *Escherichia coli* [235], thus raising the hypothesis that this compound can be released by some intestinal bacterial species and taken up by others. This compound reduces the growth of the foodborne pathogen *Listeria monocytogenes*, an effect which is associated with alteration of bacterial morphology and with decreased expression of genes known to be involved in bacterial virulence [236].

*Organic acids derived from amino acids are used as energy substrates in bacteria and can modulate bacterial growth and virulence*

During the catabolism of amino acids within bacteria, several organic acids, including succinate, oxaloacetate, formate, and lactate, represent intermediary or terminal metabolites which may be produced in significant amounts [15,237]. These compounds are obviously not produced only from amino acids but can originate from other substrates, like carbohydrates [238,239].

Succinate oxidation by *Escherichia coli* was described more than seven decades ago [240]. Since then, several studies on that topic have been performed, including a study showing capacity of *Bacillus lactis* to use succinate for respiration [241]. Succinate can be used by bacteria for ATP and reduced equivalent generation. Succinate is involved in mycobacteria respiration, serving both as an intermediate in the tricarboxylic acid cycle and as an electron donor for the respiratory chain [242]. Of note, impaired succinate oxidation in the intestinal bacterium *Mycobacterium tuberculosis* prevents its growth, thus pointing out a central role of the oxidation of this metabolite for *M. tuberculosis* proliferation. In this latter bacterium, succinate dehydrogenase, which converts succinate to fumarate, acts as a central regulator of respiration [243]. Succinate produced by gut microbiota promotes infection by the opportunistic intestinal pathogen *Clostridium difficile* [244], and extracellular succinate promotes biofilm formation by this pathogen [245]. Also of major interest, *Clostridium butyricum*, a bacterium found in the human gut that produces butyrate, can diminish the proliferation of *C. difficile* by decreasing the succinate concentration within the large intestine luminal fluid [246]. Such a decrease in the succinate concentration apparently represents the net output of the overall metabolic activity of the intestinal microbiota, suggesting subtle metabolic relationships between intestinal bacteria.

The tricarboxylic acid cycle intermediate oxaloacetate, when produced by *Escherichia coli*, can improve the survival of the amoeba parasite *Entamoeba histolytica* in the large intestine luminal fluid [247]. This result suggests bacteria–parasite communication between microorganisms in the large intestine. *Entamoeba histolytica* is the causative agent of human amoebiasis, an enteropathy affecting millions of humans worldwide [248].

Regarding formate, this organic acid is involved in energy metabolism in different bacterial species. Formate enhances respiration in the intestinal bacterial species *Campylobacter jejuni* [249]. However, at high concentrations, formate can reduce the growth of several sulfate-reducing bacteria [250], notably the species *Desulfovibrio vulgaris* found in the human intestine. Formate has been demonstrated to be secreted by the intestinal pathogen *Shigella flexneri*, promoting the expression of genes involved in its own virulence [251].

Lactic acid bacteria ferment several substrates predominantly to lactate [252]. Lactate is well known to be utilized as an oxidative substrate and ATP source by many intestinal bacteria, such as *Salmonella* and *Campylobacter* [253,254].

Lastly, concerning citrate, some intestinal bacterial species, such as sulfate-reducing bacteria and *Escherichia coli*, can use several amino acids (as well as other substrates), giving rise to the synthesis of citrate during their catabolism [255,256]. Bacteria can also import citrate from extracellular sources [257]. Endogenous sources of citrate in bacteria involve citrate synthase, which converts oxaloacetate and acetyl-CoA to citrate, while importation from the extracellular media involves, as expected, citrate transporters [258,259]. Citrate metabolism has been studied in intestinal bacteria such as *Lactobacillus casei*, and the results obtained show that the major metabolic end products formed are pyruvate, lactate, and acetoin [260]. In intestinal bacteria such as *Klebsiella pneumoniae* and *Enterobacter faecium*, citrate represents an oxidative and ATP-producing substrate [257,261]. Citrate metabolism in bacteria is at the origin of the generation of a membrane potential and a pH gradient involved in ATP synthesis [262,263]. In addition to its role in bacterial energy metabolism, citrate is a precursor for the synthesis of staphyloferrins. These compounds, which belong to the family of siderophores, can stimulate bacterial growth under condition of iron restriction [264].

## 4. Conclusions and Perspectives

The idea that the host is the main supplier of amino acids for the metabolism and physiology of bacteria contained within the intestinal luminal fluid originates from the fact that significant amounts of undigested proteins, either of dietary or endogenous origin, are transferred from the ileum to the large intestine. The utilization of these proteins, after proteolysis by the intestinal bacteria, may be viewed, as done in the present review, as a form of recycling of amino acids which have not been used by the host. These amino acids can be utilized for bacterial metabolism and physiology. Indeed, numerous bacteria of the intestinal microbiota do not possess the metabolic capacity to synthesize the 22 amino acids required for protein synthesis and for utilization of specific amino acids in other metabolic pathways. Thus, these amino acids that must be available from the extracellular medium can be viewed as “bacterially indispensable”. They are crucial for the survival of the intestinal bacteria and for the maintenance of the bacterial mass within the large intestine in the context of regular excretion of bacteria in the feces.

In this paper, we have focused on the effects of the amino acid-derived metabolites which have been shown to be involved in the regulation of intestinal bacteria growth and in the bacterial metabolism and physiology. A growing number of studies have reported on the effects of such bacterial amino acid derivatives on the virulence of the intestinal bacteria, on their capacity to form biofilms, and on their ability to face changing luminal parameters, such as osmolarity, pH, and oxidative stress-generating environments (reviewed here and in [23]). The effects of amino acid-derived metabolites on bacterial respiration and energy metabolism have also been reported, and such effects have been in some cases linked with effects on bacterial growth. Also of major interest, recent papers indicate that the metabolism of amino acids within intestinal bacteria leads to the production of compounds released in the extracellular medium, and which can act on other bacterial species present in the medium [106,143,202,246], thus revealing a new mode of communication between bacteria from different species. From the few emerging results presented in this review, it appears that this type of communication is not exclusively related to communication between intestinal bacteria but is also related to communication between intestinal bacteria and other intestinal microorganisms, such as fungi and parasites [194,247].

The quantities of the amino acid precursors derived from undigested proteins available for the metabolism of bacteria in the large intestine luminal fluid likely represent a central parameter which will influence the concentrations of bioactive bacterial metabolites derived from amino acids in this fluid. In support of this concept, it has been shown in a randomized controlled double-blind clinical trial that supplementation with either plant or animal protein (with different amino acid compositions but given in the same quantity) in volunteers led to differences in the composition of the amino acid-derived bacterial metabolites in feces and urine [265].

However, some reservations must be formulated regarding the interpretation of the available results, and numerous questions still need to be answered in future experimental and clinical works. Firstly, most studies were performed in vitro with intestinal bacteria in a context that often does not fully mimic the conditions that prevail in the large intestine. Secondly, the amino acid-derived metabolites were often tested at concentrations which may not be those that are found in the human large intestine luminal fluid. Of note, the concentrations of bacterial metabolites within the large intestine luminal fluid may, for some of them, largely diverge among individuals, and more information is needed to determine the proportion of metabolites in their free (presumably bioactive) form and in forms bound to luminal compounds. In addition, the intracellular concentrations of metabolites in intestinal bacteria which are associated with bioactive effects remain unknown in almost all cases. Third, these amino acid derivatives were generally tested individually, thus not representing the complex mixture of these derivatives as found in the large intestine content in real-life conditions.

With these reservations in mind, we are facing an exciting and stimulating area of research and future works should help to better understand the way amino acid metabolism within intestinal bacteria influences their growth and physiology. We can presume that such modifications may in turn change the composition of the intestinal microbiota in terms of commensal and pathogenic microorganisms. Such additional works will certainly provide precious indications which will prove to be useful in terms of human health from both preventive and curative perspectives.

## Data Availability

No new data were created or analyzed in this study. Data sharing is not applicable to this article.

## References

[1] Blachier F. (2023). Metabolism of Alimentary Compounds by the Intestinal Microbiota and Health.

[2] Schleifer K.H. (2009). Classification of bacteria and archaea: Past, present and future. Syst. Appl. Microbiol..

[3] Matijašić M., Meštrović T., Paljetak H.Č., Perić M., Barešić A., Verbanac D. (2020). Gut microbiota beyond bacteria-mycobiome, virome, archaeome, and eukaryotic parasites in IBD. Int. J. Mol. Sci..

[4] Rigottier-Gois L. (2013). Dysbiosis in inflammatory bowel diseases: The oxygen hypothesis. ISME J..

[5] Carding S.R., Davis N., Hoyles L. (2017). Review article: The human intestinal virome in health and disease. Aliment. Pharmacol. Ther..

[6] Sausset R., Petit M.A., Gaboriau-Routhiau V., De Paepe M. (2020). New insights into intestinal phages. Mucosal Immunol..

[7] Sartor R.B., Wu G.D. (2017). Roles for Intestinal Bacteria, Viruses, and Fungi in Pathogenesis of Inflammatory Bowel Diseases and Therapeutic Approaches. Gastroenterology.

[8] Burgess S.L., Gilchrist C.A., Lynn T.C., Petri W.A. (2017). Parasitic Protozoa and Interactions with the Host Intestinal Microbiota. Infect. Immun..

[9] Marteau P., Pochart P., Doré J., Béra-Maillet C., Bernalier A., Corthier G. (2001). Comparative study of bacterial groups within the human cecal and fecal microbiota. Appl. Environ. Microbiol..

[10] Stephen A.M., Cummings J.H. (1980). The microbial contribution to human faecal mass. J. Med. Microbiol..

[11] Stephen A.M., Wiggins H.S., Cummings J.H. (1987). Effect of changing transit time on colonic microbial metabolism in man. Gut.

[12] Bharucha A.E., Anderson B., Bouchoucha M. (2019). More movement with evaluating colonic transit in humans. Neurogastroenterol. Motil..

[13] Flint H.J., Scott K.P., Louis P., Duncan S.H. (2012). The role of the gut microbiota in nutrition and health. Nat. Rev. Gastroenterol. Hepatol..

[14] Winter S.E., Bäumler A.J. (2014). Why related bacterial species bloom simultaneously in the gut: Principles underlying the ‘Like will to like’ concept. Cell. Microbiol..

[15] Macfarlane G.T., Cummings J.H., Phillips S.F., Pemberton J.H., Shorter R.G. (1991). The colonic flora, fermentation, and large bowel digestive function. The Large Intestine: Physiology, Pathophysiology, and Disease.

[16] Windey K., De Preter V., Verbeke K. (2012). Relevance of protein fermentation to gut health. Mol. Nutr. Food Res..

[17] Korpela K. (2018). Diet, Microbiota, and Metabolic Health: Trade-Off Between Saccharolytic and Proteolytic Fermentation. Annu. Rev. Food Sci. Technol..

[18] Smith E.A., Macfarlane G.T. (1996). Enumeration of human colonic bacteria producing phenolic and indolic compounds: Effects of pH, carbohydrate availability and retention time on dissimilatory aromatic amino acid metabolism. J. Appl. Bacteriol..

[19] Birkett A., Muir J., Phillips J., Jones G., O’Dea K. (1996). Resistant starch lowers fecal concentrations of ammonia and phenols in humans. Am. J. Clin. Nutr..

[20] Geboes K.P., De Hertogh G., De Preter V., Luypaerts A., Bammens B., Evenepoel P., Ghoos Y., Geboes K., Rutgeerts P., Verbeke K. (2006). The influence of inulin on the absorption of nitrogen and the production of metabolites of protein fermentation in the colon. Br. J. Nutr..

[21] Cummings J.H., Hill M.J., Bone E.S., Branch W.J., Jenkins D.J. (1979). The effect of meat protein and dietary fiber on colonic function and metabolism. II. Bacterial metabolites in feces and urine. Am. J. Clin. Nutr..

[22] Macfarlane G.T., Cummings J.H., Macfarlane S., Gibson G.R. (1989). Influence of retention time on degradation of pancreatic enzymes by human colonic bacteria grown in a 3-stage continuous culture system. J. Appl. Bacteriol..

[23] Blachier F. (2025). Amino acid metabolism for bacterial physiology. The Evolutionary Journey of Amino Acids. From the Origin of Life to Human Metabolism.

[24] Bröer S. (2023). Intestinal Amino Acid Transport and Metabolic Health. Annu. Rev. Nutr..

[25] Gaudichon C., Bos C., Morens C., Petzke K.J., Mariotti F., Everwand J., Benamouzig R., Daré S., Tomé D., Metges C.C. (2002). Ileal losses of nitrogen and amino acids in humans and their importance to the assessment of amino acid requirements. Gastroenterology.

[26] Blachier F., Andriamihaja M., Kong X.F. (2021). Fate of undigested proteins in the pig large intestine: What impact on the colon epithelium?. Anim. Nutr..

[27] Baglieri A., Mahe S., Zidi S., Huneau J.F., Thuillier F., Marteau P., Tome D. (1994). Gastro-jejunal digestion of soya-bean-milk protein in humans. Br. J. Nutr..

[28] Bos C., Juillet B., Fouillet H., Turlan L., Daré S., Luengo C., N’tounda R., Benamouzig R., Gausserès N., Tomé D. (2005). Postprandial metabolic utilization of wheat protein in humans. Am. J. Clin. Nutr..

[29] Yao C.K., Muir J.G., Gibson P.R. (2016). Review article: Insights into colonic protein fermentation, its modulation and potential health implications. Aliment. Pharmacol. Ther..

[30] Gibson J.A., Sladen G.E., Dawson A.M. (1976). Protein absorption and ammonia production: The effects of dietary protein and removal of the colon. Br. J. Nutr..

[31] Kramer P. (1966). The effect of varying sodium loads on the ileal excreta of human ileostomized subjects. J. Clin. Investig..

[32] Smiddy F.G., Gregory S.D., Smith I.B., Goligher J. (1960). Faecal loss of fluid, electrolytes, and nitrogen in colitis before and after ileostomy. Lancet.

[33] Chacko A., Cummings J.H. (1988). Nitrogen losses from the human small bowel: Obligatory losses and the effect of physical form of food. Gut.

[34] Dubuisson C., Lioret S., Touvier M., Dufour A., Calamassi-Tran G., Volatier J.L., Lafay L. (2010). Trends in food and nutritional intakes of French adults from 1999 to 2007: Results from the INCA surveys. Br. J. Nutr..

[35] Pasiakos S.M., Agarwal S., Lieberman H.R., Fulgoni V.L. (2015). Sources and Amounts of Animal, Dairy, and Plant Protein Intake of US Adults in 2007–2010. Nutrients.

[36] Blachier F. (2025). Amino acid metabolism in the large intestine and physiological consequences. The Evolutionary Journey of Amino Acids. From the Origin of Life to Human Metabolism.

[37] Kaman W.E., Hays J.P., Endtz H.P., Bikker F.J. (2014). Bacterial proteases: Targets for diagnostics and therapy. Eur. J. Clin. Microbiol. Infect. Dis..

[38] Portune K., Beaumont M., Davila A.M., Tomé D., Blachier F., Sanz Y. (2016). Gut microbiota role in protein metabolism and health-related outcomes: The two side of the coin. Trends Food Sci. Technol..

[39] Pessione E. (2012). Lactic acid bacteria contribution to gut microbiota complexity: Lights and shadows. Front. Cell. Infect. Microbiol..

[40] Liu M., Bayjanov J.R., Renckens B., Nauta A., Siezen R.J. (2010). The proteolytic system of lactic acid bacteria revisited: A genomic comparison. BMC Genom..

[41] Saier M.H. (2000). Families of transmembrane transporters selective for amino acids and their derivatives. Microbiology.

[42] Tanaka K.J., Song S., Mason K., Pinkett H.W. (2018). Selective substrate uptake: The role of ATP-binding cassette (ABC) importers in pathogenesis. Biochim. Biophys. Acta Biomembr..

[43] Hosie A.H., Poole P.S. (2001). Bacterial ABC transporters of amino acids. Res. Microbiol..

[44] Burkovski A., Krämer R. (2002). Bacterial amino acid transport proteins: Occurrence, functions, and significance for biotechnological applications. Appl. Microbiol. Biotechnol..

[45] Konings W.N. (2002). The cell membrane and the struggle for life of lactic acid bacteria. Antonie Van Leeuwenhoek.

[46] Chen Y., Dinges M.M., Green A., Cramer S.E., Larive C.K., Lytle C. (2020). Absorptive transport of amino acids by the rat colon. Am. J. Physiol..

[47] van der Wielen N., Moughan P.J., Mensink M. (2017). Amino acid absorption in the large intestine of humans and porcine models. J. Nutr..

[48] Fuller M. (2012). Determination of protein and amino acid digestibility in foods including implications of gut microbial amino acid synthesis. Br. J. Nutr..

[49] Darragh A.J., Cranwell P.D., Moughan P.J. (1994). Absorption of lysine and methionine from the proximal colon of the piglet. Br. J. Nutr..

[50] Schaafsma G. (2000). The protein digestibility-corrected amino acid score. J. Nutr..

[51] Metges C.C. (2000). Contribution of microbial amino acids to amino acid homeostasis of the host. J. Nutr..

[52] Fuller M.F., Reeds P.J. (1998). Nitrogen cycling in the gut. Annu. Rev. Nutr..

[53] Niiyama M., Deguchi E., Kagota K., Namioka S. (1979). Appearance of 15N-labeled intestinal microbial amino acids in the venous blood of the pig colon. Am. J. Vet. Res..

[54] Nakanishi T., Hatanaka T., Huang W., Prasad P.D., Leibach F.H., Ganapathy M.E., Ganapathy V. (2001). Na^+^- and Cl^−^-coupled active transport of carnitine by the amino acid transporter ATB(0,+) from mouse colon expressed in HRPE cells and Xenopus oocytes. J. Physiol..

[55] Hatanaka T., Huang W., Nakanishi T., Bridges C.C., Smith S.B., Prasad P.D., Ganapathy M.E., Ganapathy V. (2002). Transport of D-serine via the amino acid transporter ATB(0,+) expressed in the colon. Biochem. Biophys. Res. Commun..

[56] Ugawa S., Sunouchi Y., Ueda T., Takahashi E., Saishin Y., Shimada S. (2001). Characterization of a mouse colonic system B(0+) amino acid transporter related to amino acid absorption in colon. Am. J. Physiol..

[57] Blachier F., Mariotti F., Huneau J.F., Tomé D. (2007). Effects of amino acid-derived luminal metabolites on the colonic epithelium and physiopathological consequences. Amino Acids.

[58] James P.S., Smith M.W. (1976). Methionine transport by pig colonic mucosa measured during early post-natal development. J. Physiol..

[59] Sepúlveda F.V., Smith M.W. (1979). Different mechanisms for neutral amino acid uptake by new-born pig colon. J. Physiol..

[60] Hou Y., Yin Y., Wu G. (2015). Dietary essentiality of “nutritionally non-essential amino acids” for animals and humans. Exp. Biol. Med..

[61] Mathai J.K., Liu Y., Stein H.H. (2017). Values for digestible indispensable amino acid scores (DIAAS) for some dairy and plant proteins may better describe protein quality than values calculated using the concept for protein digestibility-corrected amino acid scores (PDCAAS). Br. J. Nutr..

[62] Detzner J., Pohlentz G., Müthing J. (2022). Enterohemorrhagic *Escherichia coli* and a fresh view on Shiga toxin-binding glycosphingolipids of primary human kidney and colon epithelial cells and their toxin susceptibility. Int. J. Mol. Sci..

[63] Zhang Y.G., Singhal M., Lin Z., Manzella C., Kumar A., Alrefai W.A., Dudeja P.K., Saksena S., Sun J., Gill R.K. (2018). Infection with enteric pathogens Salmonella typhimurium and *Citrobacter rodentium* modulate TGF-beta/Smad signaling pathways in the intestine. Gut Microbes.

[64] Jones S.E., Knight K.L. (2012). Bacillus subtilis-mediated protection from *Citrobacter rodentium*-associated enteric disease requires espH and functional flagella. Infect. Immun..

[65] Shrestha A., Mehdizadeh Gohari I., Li J., Navarro M., Uzal F.A., McClane B.A. (2024). The biology and pathogenicity of Clostridium perfringens type F: A common human enteropathogen with a new(ish) name. Microbiol. Mol. Biol. Rev..

[66] Shimizu T., Ohtani K., Hirakawa H., Ohshima K., Yamashita A., Shiba T., Ogasawara N., Hattori M., Kuhara S., Hayashi H. (2002). Complete genome sequence of *Clostridium perfringens*, an anaerobic flesh-eater. Proc. Natl. Acad. Sci. USA.

[67] Denou E., Rezzonico E., Panoff J.M., Arigoni F., Brüssow H. (2009). A Mesocosm of *Lactobacillus johnsonii*, *Bifidobacterium longum*, and *Escherichia coli* in the mouse gut. DNA Cell. Biol..

[68] Pridmore R.D., Berger B., Desiere F., Vilanova D., Barretto C., Pittet A.C., Zwahlen M.C., Rouvet M., Altermann E., Barrangou R. (2004). The genome sequence of the probiotic intestinal bacterium *Lactobacillus johnsonii* NCC 533. Proc. Natl. Acad. Sci. USA.

[69] Young K.T., Davis L.M., Dirita V.J. (2007). *Campylobacter jejuni*: Molecular biology and pathogenesis. Nat. Rev. Microbiol..

[70] Fan T.J., Goeser L., Naziripour A., Redinbo M.R., Hansen J.J. (2019). Enterococcus faecalis gluconate phosphotransferase system accelerates experimental colitis and bacterial killing by macrophages. Infect. Immun..

[71] Yu X.J., Walker D.H., Liu Y., Zhang L. (2009). Amino acid biosynthesis deficiency in bacteria associated with human and animal hosts. Infect. Genet. Evol..

[72] Gomes-Santos A.C., de Oliveira R.P., Moreira T.G., Castro-Junior A.B., Horta B.C., Lemos L., de Almeida L.A., Rezende R.M., Cara D.C., Oliveira S.C. (2017). Hsp65-producing *Lactococcus lactis* prevents inflammatory intestinal disease in mice by IL-10- and TLR2-dependent pathways. Front. Immunol..

[73] Bolotin A., Wincker P., Mauger S., Jaillon O., Malarme K., Weissenbach J., Ehrlich S.D., Sorokin A. (2001). The complete genome sequence of the lactic acid bacterium *Lactococcus lactis* ssp. lactis IL1403. Genome Res..

[74] Godon J.J., Delorme C., Bardowski J., Chopin M.C., Ehrlich S.D., Renault P. (1993). Gene inactivation in *Lactococcus lactis*: Branched-chain amino acid biosynthesis. J. Bacteriol..

[75] Alsultan A., Walton G., Andrews S.C., Clarke S.R. (2023). *Staphylococcus aureus* FadB is a dehydrogenase that mediates cholate resistance and survival under human colonic conditions. Microbiology.

[76] Kuroda M., Ohta T., Uchiyama I., Baba T., Yuzawa H., Kobayashi I., Cui L., Oguchi A., Aoki K., Nagai Y. (2001). Whole genome sequencing of meticillin-resistant *Staphylococcus aureus*. Lancet.

[77] Njenga R., Boele J., Öztürk Y., Koch H.G. (2023). Coping with stress: How bacteria fine-tune protein synthesis and protein transport. J. Biol. Chem..

[78] Tollerson R., Ibba M. (2020). Translational regulation of environmental adaptation in bacteria. J. Biol. Chem..

[79] Macek B., Forchhammer K., Hardouin J., Weber-Ban E., Grangeasse C., Mijakovic I. (2019). Protein post-translational modifications in bacteria. Nat. Rev. Microbiol..

[80] Hu Y., Qing Y., Chen J., Liu C., Lu J., Wang Q., Zhen S., Zhou H., Huang L., Zhang R. (2021). Prevalence, risk factors, and molecular epidemiology of intestinal carbapenem-resistant *Pseudomonas aeruginosa*. Microbiol. Spectr..

[81] Zhang J., Hoedt E.C., Liu Q., Berendsen E., Teh J.J., Hamilton A., O’ Brien A.W., Ching J.Y.L., Wei H., Yang K. (2021). Elucidation of Proteus mirabilis as a key bacterium in Crohn’s disease inflammation. Gastroenterology.

[82] Charlier D., Nguyen Le Minh P., Roovers M. (2018). Regulation of carbamoylphosphate synthesis in *Escherichia coli*: An amazing metabolite at the crossroad of arginine and pyrimidine biosynthesis. Amino Acids..

[83] Leiva L.E., Zegarra V., Bange G., Ibba M. (2023). At the crossroad of nucleotide dynamics and protein synthesis in bacteria. Microbiol. Mol. Biol. Rev..

[84] Oliphant K., Allen-Vercoe E. (2019). Macronutrient metabolism by the human gut microbiome: Major fermentation by-products and their impact on host health. Microbiome.

[85] Davila A.M., Blachier F., Gotteland M., Andriamihaja M., Benetti P.H., Sanz Y., Tomé D. (2013). Intestinal luminal nitrogen metabolism: Role of the gut microbiota and consequences for the host. Pharmacol. Res..

[86] Kim J., Hetzel M., Boiangiu C.D., Buckel W. (2004). Dehydration of (R)-2-hydroxyacyl-CoA to enoyl-CoA in the fermentation of alpha-amino acids by anaerobic bacteria. FEMS Microbiol. Rev..

[87] Buckel W. (2021). Energy conservation in fermentations of anaerobic bacteria. Front. Microbiol..

[88] Macfarlane G.T., Macfarlane S. (2012). Bacteria, colonic fermentation, and gastrointestinal health. J. AOAC Int..

[89] Barker H.A. (1981). Amino acid degradation by anaerobic bacteria. Annu. Rev. Biochem..

[90] Pessione A., Lamberti C., Pessione E. (2010). Proteomics as a tool for studying energy metabolism in lactic acid bacteria. Mol. Biosyst..

[91] Fernández M., Zúñiga M. (2006). Amino acid catabolic pathways of lactic acid bacteria. Crit. Rev. Microbiol..

[92] Pereira C.I., Matos D., San Romão M.V., Crespo M.T. (2009). Dual role for the tyrosine decarboxylation pathway in Enterococcus faecium E17: Response to an acid challenge and generation of a proton motive force. Appl. Environ. Microbiol..

[93] Stickland L.H. (1935). Studies in the metabolism of the strict anaerobes (Genus Clostridium): The reduction of proline by *Cl. sporogenes*. Biochem. J..

[94] Marshall A., McGrath J.W., Graham R., McMullan G. (2023). Food for thought-The link between *Clostridioides difficile* metabolism and pathogenesis. PLoS Pathog..

[95] Pruss K.M., Enam F., Battaglioli E., DeFeo M., Diaz O.R., Higginbottom S.K., Fischer C.R., Hryckowian A.J., Van Treuren W., Dodd D. (2022). Oxidative ornithine metabolism supports non-inflammatory *C. difficile* colonization. Nat. Metab..

[96] Pavao A., Graham M., Arrieta-Ortiz M.L., Immanuel S.R.C., Baliga N.S., Bry L. (2022). Reconsidering the in vivo functions of Clostridial Stickland amino acid fermentations. Anaerobe.

[97] Fonknechten N., Chaussonnerie S., Tricot S., Lajus A., Andreesen J.R., Perchat N., Pelletier E., Gouyvenoux M., Barbe V., Salanoubat M. (2010). *Clostridium sticklandii*, a specialist in amino acid degradation: Revisiting its metabolism through its genome sequence. BMC Genom..

[98] Blachier F. (2023). Amino acid-derived bacterial metabolites in the colorectal luminal fluid: Effects on microbial communication, metabolism, physiology, and growth. Microorganisms.

[99] Michael A.J. (2016). Polyamines in eukaryotes, bacteria, and archaea. J. Biol. Chem..

[100] Li B., Baniasadi H.R., Liang J., Phillips M.A., Michael A.J. (2025). New routes for spermine biosynthesis. J. Biol. Chem..

[101] Shah P., Swiatlo E. (2008). A multifaceted role for polyamines in bacterial pathogens. Mol. Microbiol..

[102] Yoshida M., Kashiwagi K., Shigemasa A., Taniguchi S., Yamamoto K., Makinoshima H., Ishihama A., Igarashi K. (2004). A unifying model for the role of polyamines in bacterial cell growth, the polyamine modulon. J. Biol. Chem..

[103] Chattopadhyay M.K., Keembiyehetty C.N., Chen W., Tabor H. (2015). Polyamines stimulate the level of the sigma38 subunit (RpoS) of *Escherichia coli* RNA polymerase, resulting in the induction of the glutamate decarboxylase-dependent acid response system via the gadE regulon. J. Biol. Chem..

[104] Igarashi K., Kashiwagi K. (2018). Effects of polyamines on protein synthesis and growth of *Escherichia coli*. J. Biol. Chem..

[105] Igarashi K., Kashiwagi K. (1999). Polyamine transport in bacteria and yeast. Biochem. J..

[106] Driessen A.J., Smid E.J., Konings W.N. (1988). Transport of diamines by *Enterococcus faecalis* is mediated by an agmatine-putrescine antiporter. J. Bacteriol..

[107] Large P.J. (1992). Enzymes and pathways of polyamine breakdown in microorganisms. FEMS Microbiol. Rev..

[108] Chattopadhyay M.K., Tabor C.W., Tabor H. (2009). Polyamines are not required for aerobic growth of *Escherichia coli*: Preparation of a strain with deletions in all of the genes for polyamine biosynthesis. J. Bacteriol..

[109] Nakada Y., Itoh Y. (2003). Identification of the putrescine biosynthetic genes in *Pseudomonas aeruginosa* and characterization of agmatine deiminase and N-carbamoylputrescine amidohydrolase of the arginine decarboxylase pathway. Microbiology.

[110] Hanfrey C.C., Pearson B.M., Hazeldine S., Lee J., Gaskin D.J., Woster P.M., Phillips M.A., Michael A.J. (2011). Alternative spermidine biosynthetic route is critical for growth of *Campylobacter jejuni* and is the dominant polyamine pathway in human gut microbiota. J. Biol. Chem..

[111] Chagneau C.V., Garcie C., Bossuet-Greif N., Tronnet S., Brachmann A.O., Piel J., Nougayrède J.P., Martin P., Oswald E. (2019). The polyamine spermidine modulates the production of the bacterial genotoxin colibactin. mSphere.

[112] Goforth J.B., Walter N.E., Karatan E. (2013). Effects of polyamines on *Vibrio cholerae* virulence properties. PLoS ONE..

[113] Kramer J., Özkaya Ö., Kümmerli R. (2020). Bacterial siderophores in community and host interactions. Nat. Rev. Microbiol..

[114] Hamana K., Saito T., Okada M., Sakamoto A., Hosoya R. (2002). Covalently linked polyamines in the cell wall peptidoglycan of *Selenomonas*, *Anaeromusa*, *Dendrosporobacter*, *Acidaminococcus* and *Anaerovibrio* belonging to the *Sporomusa subbranch*. J. Gen. Appl. Microbiol..

[115] Samartzidou H., Mehrazin M., Xu Z., Benedik M.J., Delcour A.H. (2003). Cadaverine inhibition of porin plays a role in cell survival at acidic pH. J. Bacteriol..

[116] Tanaka Y., Kimura B., Takahashi H., Watanabe T., Obata H., Kai A., Morozumi S., Fujii T. (2008). Lysine decarboxylase of *Vibrio parahaemolyticus*: Kinetics of transcription and role in acid resistance. J. Appl. Microbiol..

[117] Hall-Stoodley L., Costerton J.W., Stoodley P. (2004). Bacterial biofilms: From the natural environment to infectious diseases. Nat. Rev. Microbiol..

[118] Flemming H.C., Wingender J. (2010). The biofilm matrix. Nat. Rev. Microbiol..

[119] Blachier F., Beaumont M., Andriamihaja M., Davila A.M., Lan A., Grauso M., Armand L., Benamouzig R., Tomé D. (2017). Changes in the luminal environment of the colonic epithelial cells and physiopathological consequences. Am. J. Pathol..

[120] Probert H.M., Gibson G.R. (2002). Bacterial biofilms in the human gastrointestinal tract. Curr. Issues Intest. Microbiol..

[121] Roy R., Tiwari M., Donelli G., Tiwari V. (2018). Strategies for combating bacterial biofilms: A focus on anti-biofilm agents and their mechanisms of action. Virulence.

[122] Solano C., Echeverz M., Lasa I. (2014). Biofilm dispersion and quorum sensing. Curr. Opin. Microbiol..

[123] Mukherjee S., Bassler B.L. (2019). Bacterial quorum sensing in complex and dynamically changing environments. Nat. Rev. Microbiol..

[124] Banerji R., Kanojiya P., Saroj S.D. (2020). Role of interspecies bacterial communication in the virulence of pathogenic bacteria. Crit. Rev. Microbiol..

[125] Burrell M., Hanfrey C.C., Murray E.J., Stanley-Wall N.R., Michael A.J. (2010). Evolution and multiplicity of arginine decarboxylases in polyamine biosynthesis and essential role in *Bacillus subtilis* biofilm formation. J. Biol. Chem..

[126] Prentice J.A., Bridges A.A., Bassler B.L. (2022). Synergy between c-di-GMP and quorum-sensing signaling in *Vibrio cholerae* biofilm morphogenesis. J. Bacteriol..

[127] Karatan E., Duncan T.R., Watnick P.I. (2005). NspS, a predicted polyamine sensor, mediates activation of *Vibrio cholerae* biofilm formation by norspermidine. J. Bacteriol..

[128] Lee J., Sperandio V., Frantz D.E., Longgood J., Camilli A., Phillips M.A., Michael A.J. (2009). An alternative polyamine biosynthetic pathway is widespread in bacteria and essential for biofilm formation in *Vibrio cholerae*. J. Biol. Chem..

[129] Sobe R.C., Bond W.G., Wotanis C.K., Zayner J.P., Burriss M.A., Fernandez N., Bruger E.L., Waters C.M., Neufeld H.S., Karatan E. (2017). Spermine inhibits *Vibrio cholerae* biofilm formation through the NspS-MbaA polyamine signaling system. J. Biol. Chem..

[130] Blachier F., Davila A.M., Mimoun S., Benetti P.H., Atanasiu C., Andriamihaja M., Benamouzig R., Bouillaud F., Tomé D. (2010). Luminal sulfide and large intestine mucosa: Friend or foe?. Amino Acids.

[131] Barton L.L., Ritz N.L., Fauque G.D., Lin H.C. (2017). Sulfur cycling and the intestinal microbiome. Dig. Dis. Sci..

[132] Basic A., Blomqvist M., Dahlén G., Svensäter G. (2017). The proteins of *Fusobacterium* spp. involved in hydrogen sulfide production from L-cysteine. BMC Microbiol..

[133] Linden D.R. (2014). Hydrogen sulfide signaling in the gastrointestinal tract. Antioxid. Redox Signal..

[134] Croix J.A., Carbonero F., Nava G.M., Russell M., Greenberg E., Gaskins H.R. (2011). On the relationship between sialomucin and sulfomucin expression and hydrogenotrophic microbes in the human colonic mucosa. PLoS ONE.

[135] Villanueva-Millan M.J., Leite G., Mathur R., Rezaie A., Fajardo C.M., de Freitas Germano J., Morales W., Sanchez M., Rivera I., Parodi G. (2025). Hydrogen Sulfide and Methane on Breath Test Correlate with Human Small Intestinal Hydrogen Sulfide Producers and Methanogens. Dig. Dis. Sci..

[136] Gibson G.R., Macfarlane G.T., Cummings J.H. (1988). Occurrence of sulphate-reducing bacteria in human faeces and the relationship of dissimilatory sulphate reduction to methanogenesis in the large gut. J. Appl. Bacteriol..

[137] Ohge H., Furne J.K., Springfield J., Sueda T., Madoff R.D., Levitt M.D. (2003). The effect of antibiotics and bismuth on fecal hydrogen sulfide and sulfate-reducing bacteria in the rat. FEMS Microbiol. Lett..

[138] Rowan F.E., Docherty N.G., Coffey J.C., O’Connell P.R. (2009). Sulphate-reducing bacteria and hydrogen sulphide in the aetiology of ulcerative colitis. Br. J. Surg..

[139] Kushkevych I., Dordević D., Alberfkani M.I., Gajdács M., Ostorházi E., Vítězová M., Rittmann S.K.R. (2023). NADH and NADPH peroxidases as antioxidant defense mechanisms in intestinal sulfate-reducing bacteria. Sci. Rep..

[140] Florin T., Neale G., Gibson G.R., Christl S.U., Cummings J.H. (1991). Metabolism of dietary sulphate: Absorption and excretion in humans. Gut.

[141] Lewis S., Cochrane S. (2007). Alteration of sulfate and hydrogen metabolism in the human colon by changing intestinal transit rate. Am. J. Gastroenterol..

[142] Blachier F., Andriamihaja M., Larraufie P., Ahn E., Lan A., Kim E. (2021). Production of hydrogen sulfide by the intestinal microbiota and epithelial cells and consequences for the colonic and rectal mucosa. Am. J. Physiol..

[143] Bouillaud F., Blachier F. (2011). Mitochondria and sulfide: A very old story of poisoning, feeding, and signaling?. Antioxid. Redox Signal..

[144] Jørgensen J., Mortensen P.B. (2001). Hydrogen sulfide and colonic epithelial metabolism: Implications for ulcerative colitis. Dig. Dis. Sci..

[145] Suarez F., Furne J., Springfield J., Levitt M. (1998). Production and elimination of sulfur-containing gases in the rat colon. Am. J. Physiol..

[146] Jensen B., Fago A. (2018). Reactions of ferric hemoglobin and myoglobin with hydrogen sulfide under physiological conditions. J. Inorg. Biochem..

[147] Andriamihaja M., Lan A., Beaumont M., Grauso M., Gotteland M., Pastene E., Cires M.J., Carrasco-Pozo C., Tomé D., Blachier F. (2018). Proanthocyanidin-containing polyphenol extracts from fruits prevent the inhibitory effect of hydrogen sulfide on human colonocyte oxygen consumption. Amino Acids.

[148] Oldham K.E.A., Prentice E.J., Summers E.L., Hicks J.L. (2022). Serine acetyltransferase from *Neisseria gonorrhoeae*; structural and biochemical basis of inhibition. Biochem. J..

[149] Hicks J.L., Mullholland C.V. (2018). Cysteine biosynthesis in Neisseria species. Microbiology.

[150] Yao S., Zhao Z., Wang W., Liu X. (2021). *Bifidobacterium longum*: Protection against inflammatory bowel disease. J. Immunol. Res..

[151] Schell M.A., Karmirantzou M., Snel B., Vilanova D., Berger B., Pessi G., Zwahlen M.C., Desiere F., Bork P., Delley M. (2002). The genome sequence of *Bifidobacterium longum* reflects its adaptation to the human gastrointestinal tract. Proc. Natl. Acad. Sci. USA.

[152] Rodionov D.A., Vitreschak A.G., Mironov A.A., Gelfand M.S. (2004). Comparative genomics of the methionine metabolism in Gram-positive bacteria: A variety of regulatory systems. Nucleic Acids Res..

[153] Ferla M.P., Patrick W.M. (2014). Bacterial methionine biosynthesis. Microbiology.

[154] Saini V., Chinta K.C., Reddy V.P., Glasgow J.N., Stein A., Lamprecht D.A., Rahman M.A., Mackenzie J.S., Truebody B.E., Adamson J.H. (2020). Hydrogen sulfide stimulates *Mycobacterium tuberculosis* respiration, growth and pathogenesis. Nat. Commun..

[155] Borisov V.B., Forte E. (2021). Impact of hydrogen sulfide on mitochondrial and bacterial bioenergetics. Int. J. Mol. Sci..

[156] Melo A.M., Teixeira M. (2016). Supramolecular organization of bacterial aerobic respiratory chains: From cells and back. Biochim. Biophys. Acta..

[157] Forte E., Borisov V.B., Falabella M., Colaço H.G., Tinajero-Trejo M., Poole R.K., Vicente J.B., Sarti P., Giuffrè A. (2016). The terminal oxidase cytochrome bd promotes sulfide-resistant bacterial respiration and growth. Sci. Rep..

[158] Nastasi M.R., Caruso L., Giordano F., Mellini M., Rampioni G., Giuffrè A., Forte E. (2024). Cyanide insensitive oxidase confers hydrogen sulfide and nitric oxide tolerance to *Pseudomonas aeruginosa* aerobic respiration. Antioxidants.

[159] Bachenheimer A.G., Bennett E.O. (1961). The sensitivity of mixed population of bacteria to inhibitors. I. The mechanism by which *Desulfovibrio desulfuricans* protects *Ps. aeruginosa* from the toxicity of mercurials. Antonie Van Leeuwenhoek.

[160] Stutzenberger F.J., Bennett E.O. (1965). Sensitivity of mixed populations of Staphylococcus aureus and *Escherichia coli* to mercurials. Appl. Microbiol..

[161] Shatalin K., Shatalina E., Mironov A., Nudler E. (2011). H2S: A universal defense against antibiotics in bacteria. Science.

[162] Pal V.K., Bandyopadhyay P., Singh A. (2018). Hydrogen sulfide in physiology and pathogenesis of bacteria and viruses. IUBMB Life.

[163] Mironov A., Seregina T., Nagornykh M., Luhachack L.G., Korolkova N., Lopes L.E., Kotova V., Zavilgelsky G., Shakulov R., Shatalin K. (2017). Mechanism of H_2_S-mediated protection against oxidative stress in *Escherichia coli*. Proc. Natl. Acad. Sci. USA.

[164] Shukla P., Khodade V.S., SharathChandra M., Chauhan P., Mishra S., Siddaramappa S., Pradeep B.E., Singh A., Chakrapani H. (2017). “On demand” redox buffering by H_2_S contributes to antibiotic resistance revealed by a bacteria-specific H_2_S donor. Chem. Sci..

[165] Shatalin K., Nuthanakanti A., Kaushik A., Shishov D., Peselis A., Shamovsky I., Pani B., Lechpammer M., Vasilyev N., Shatalina E. (2021). Inhibitors of bacterial H_2_S biogenesis targeting antibiotic resistance and tolerance. Science.

[166] Ng S.Y., Ong K.X., Surendran S.T., Sinha A., Lai J.J.H., Chen J., Liang J., Tay L.K.S., Cui L., Loo H.L. (2020). Hydrogen Sulfide Sensitizes Acinetobacter baumannii to Killing by Antibiotics. Front. Microbiol..

[167] Stacy A., Andrade-Oliveira V., McCulloch J.A., Hild B., Oh J.H., Perez-Chaparro P.J., Sim C.K., Lim A.I., Link V.M., Enamorado M. (2021). Infection trains the host for microbiota-enhanced resistance to pathogens. Cell.

[168] Motta J.P., Flannigan K.L., Agbor T.A., Beatty J.K., Blackler R.W., Workentine M.L., Da Silva G.J., Wang R., Buret A.G., Wallace J.L. (2015). Hydrogen sulfide protects from colitis and restores intestinal microbiota biofilm and mucus production. Inflamm. Bowel Dis..

[169] Chen Y.W., Camacho M.I., Chen Y., Bhat A.H., Chang C., Peluso E.A., Wu C., Das A., Ton-That H. (2022). Genetic determinants of hydrogen sulfide biosynthesis in *Fusobacterium nucleatum* are required for bacterial fitness, antibiotic sensitivity, and virulence. mBio.

[170] Sun J., Wang X., Gao Y., Li S., Hu Z., Huang Y., Fan B., Wang X., Liu M., Qiao C. (2024). H_2_S scavenger as a broad-spectrum strategy to deplete bacteria-derived H_2_S for antibacterial sensitization. Nat. Commun..

[171] Sudhamsu J., Crane B.R. (2009). Bacterial nitric oxide synthases: What are they good for?. Trends Microbiol..

[172] Adak S., Aulak K.S., Stuehr D.J. (2002). Direct evidence for nitric oxide production by a nitric-oxide synthase-like protein from Bacillus subtilis. J. Biol. Chem..

[173] Morita H., Yoshikawa H., Sakata R., Nagata Y., Tanaka H. (1997). Synthesis of nitric oxide from the two equivalent guanidino nitrogens of L-arginine by *Lactobacillus fermentum*. J. Bacteriol..

[174] Sellars M.J., Hall S.J., Kelly D.J. (2002). Growth of *Campylobacter jejuni* supported by respiration of fumarate, nitrate, nitrite, trimethylamine-N-oxide, or dimethyl sulfoxide requires oxygen. J. Bacteriol..

[175] Weingarten R.A., Grimes J.L., Olson J.W. (2008). Role of *Campylobacter jejuni* respiratory oxidases and reductases in host colonization. Appl. Environ. Microbiol..

[176] Silvestrini M.C., Falcinelli S., Ciabatti I., Cutruzzolà F., Brunori M. (1994). *Pseudomonas aeruginosa* nitrite reductase (or cytochrome oxidase): An overview. Biochimie.

[177] Daims H., Lebedeva E.V., Pjevac P., Han P., Herbold C., Albertsen M., Jehmlich N., Palatinszky M., Vierheilig J., Bulaev A. (2015). Complete nitrification by *Nitrospira bacteria*. Nature.

[178] Zheng Y., Ke J., Song J., Li X., Kuang R., Wang H., Li S., Li Y. (2024). Correlation between daily physical activity and intestinal microbiota in perimenopausal women. Sports Med. Health Sci..

[179] Kastner J., Pfeffel F., Rajek A., Pezawas T., Hiesmayr M., Eichler H.G. (1997). Nitric oxide concentration in the gas phase of the gastrointestinal tract in man. Eur. J. Clin. Investig..

[180] Richardson A.R., Payne E.C., Younger N., Karlinsey J.E., Thomas V.C., Becker L.A., Navarre W.W., Castor M.E., Libby S.J., Fang F.C. (2011). Multiple targets of nitric oxide in the tricarboxylic acid cycle of *Salmonella enterica* serovar typhimurium. Cell Host Microbe.

[181] Stern A.M., Zhu J. (2014). An introduction to nitric oxide sensing and response in bacteria. Adv. Appl. Microbiol..

[182] Zhao G., Guo Y.Y., Yao S., Shi X., Lv L., Du Y.L. (2020). Nitric oxide as a source for bacterial triazole biosynthesis. Nat. Commun..

[183] Caranto J.D. (2019). The emergence of nitric oxide in the biosynthesis of bacterial natural products. Curr. Opin. Chem. Biol..

[184] Choules M.P., Wolf N.M., Lee H., Anderson J.R., Grzelak E.M., Wang Y., Ma R., Gao W., McAlpine J.B., Jin Y.Y. (2019). Rufomycin targets ClpC1 proteolysis in *Mycobacterium tuberculosis* and *M. abscessus*. Antimicrob. Agents Chemother..

[185] Anantharaman S., Guercio D., Mendoza A.G., Withorn J.M., Boon E.M. (2023). Negative regulation of biofilm formation by nitric oxide sensing proteins. Biochem. Soc. Trans..

[186] Poh W.H., Rice S.A. (2022). Recent Developments in nitric oxide donors and delivery for antimicrobial and anti-biofilm applications. Molecules.

[187] Ueno T., Fischer J.T., Boon E.M. (2019). Nitric oxide enters quorum sensing via the H-NOX signaling pathway in *Vibrio parahaemolyticus*. Front. Microbiol..

[188] Keszthelyi D., Troost F.J., Masclee A.A. (2009). Understanding the role of tryptophan and serotonin metabolism in gastrointestinal function. Neurogastroenterol. Motil..

[189] Lee J.H., Wood T.K., Lee J. (2015). Roles of indole as an interspecies and interkingdom signaling molecule. Trends Microbiol..

[190] Roager H.M., Licht T.R. (2018). Microbial tryptophan catabolites in health and disease. Nat. Commun..

[191] Rattanaphan P., Mittraparp-Arthorn P., Srinoun K., Vuddhakul V., Tansila N. (2020). Indole signaling decreases biofilm formation and related virulence of *Listeria monocytogenes*. FEMS Microbiol. Lett..

[192] Lee J., Attila C., Cirillo S.L., Cirillo J.D., Wood T.K. (2009). Indole and 7-hydroxyindole diminish *Pseudomonas aeruginosa* virulence. Microb. Biotechnol..

[193] Nikaido E., Giraud E., Baucheron S., Yamasaki S., Wiedemann A., Okamoto K., Takagi T., Yamaguchi A., Cloeckaert A., Nishino K. (2012). Effects of indole on drug resistance and virulence of *Salmonella enterica* serovar Typhimurium revealed by genome-wide analyses. Gut Pathog..

[194] Oh S., Go G.W., Mylonakis E., Kim Y. (2012). The bacterial signalling molecule indole attenuates the virulence of the fungal pathogen *Candida albicans*. J. Appl. Microbiol..

[195] Talapko J., Juzbašić M., Matijević T., Pustijanac E., Bekić S., Kotris I., Škrlec I. (2021). *Candida albicans*-The virulence factors and clinical manifestations of infection. J. Fungi.

[196] Nowak A., Libudzisz Z. (2006). Influence of phenol, p-cresol and indole on growth and survival of intestinal lactic acid bacteria. Anaerobe.

[197] Ledala N., Malik M., Rezaul K., Paveglio S., Provatas A., Kiel A., Caimano M., Zhou Y., Lindgren J., Krasulova K. (2022). Bacterial indole as a multifunctional regulator of *Klebsiella oxytoca* complex enterotoxicity. mBio.

[198] Högenauer C., Langner C., Beubler E., Lippe I.T., Schicho R., Gorkiewicz G., Krause R., Gerstgrasser N., Krejs G.J., Hinterleitner T.A. (2006). *Klebsiella oxytoca* as a causative organism of antibiotic-associated hemorrhagic colitis. N. Engl. J. Med..

[199] Bone E., Tamm A., Hill M. (1976). The production of urinary phenols by gut bacteria and their possible role in the causation of large bowel cancer. Am. J. Clin. Nutr..

[200] Saito Y., Sato T., Nomoto K., Tsuji H. (2018). Identification of phenol- and p-cresol-producing intestinal bacteria by using media supplemented with tyrosine and its metabolites. FEMS Microbiol. Ecol..

[201] Harrison M.A., Kaur H., Wren B.W., Dawson L.F. (2021). Production of p-cresol by Decarboxylation of p-HPA by All Five Lineages of *Clostridioides difficile* Provides a Growth Advantage. Front. Cell. Infect. Microbiol..

[202] Passmore I.J., Letertre M.P.M., Preston M.D., Bianconi I., Harrison M.A., Nasher F., Kaur H., Hong H.A., Baines S.D., Cutting S.M. (2018). Para-cresol production by Clostridium difficile affects microbial diversity and membrane integrity of Gram-negative bacteria. PLoS Pathog..

[203] Abt M.C., McKenney P.T., Pamer E.G. (2016). Clostridium difficile colitis: Pathogenesis and host defence. Nat. Rev. Microbiol..

[204] Hafiz S., Oakley C.L. (1976). *Clostridium difficile*: Isolation and characteristics. J. Med. Microbiol..

[205] Dawson L.F., Donahue E.H., Cartman S.T., Barton R.H., Bundy J., McNerney R., Minton N.P., Wren B.W. (2011). The analysis of para-cresol production and tolerance in *Clostridium difficile* 027 and 012 strains. BMC Microbiol..

[206] Jensen M.T., Cox R.P., Jensen B.B. (1995). 3-Methylindole (skatole) and indole production by mixed populations of pig fecal bacteria. Appl. Environ. Microbiol..

[207] Yokoyama M.T., Carlson J.R. (1979). Microbial metabolites of tryptophan in the intestinal tract with special reference to skatole. Am. J. Clin. Nutr..

[208] Whitehead T.R., Price N.P., Drake H.L., Cotta M.A. (2008). Catabolic pathway for the production of skatole and indoleacetic acid by the acetogen *Clostridium drakei*, *Clostridium scatologenes*, and swine manure. Appl. Environ. Microbiol..

[209] Choi S.H., Kim Y., Oh S., Oh S., Chun T., Kim S.H. (2014). Inhibitory effect of skatole (3-methylindole) on enterohemorrhagic Escherichia coli O157:H7 ATCC 43894 biofilm formation mediated by elevated endogenous oxidative stress. Lett. Appl. Microbiol..

[210] Morbach S., Krämer R. (2005). Structure and function of the betaine uptake system BetP of *Corynebacterium glutamicum*: Strategies to sense osmotic and chill stress. J. Mol. Microbiol. Biotechnol..

[211] Nau-Wagner G., Opper D., Rolbetzki A., Boch J., Kempf B., Hoffmann T., Bremer E. (2012). Genetic control of osmoadaptive glycine betaine synthesis in *Bacillus subtilis* through the choline-sensing and glycine betaine-responsive GbsR repressor. J. Bacteriol..

[212] Zou H., Chen N., Shi M., Xian M., Song Y., Liu J. (2016). The metabolism and biotechnological application of betaine in microorganism. Appl. Microbiol. Biotechnol..

[213] Wargo M.J. (2013). Homeostasis and catabolism of choline and glycine betaine: Lessons from *Pseudomonas aeruginosa*. Appl. Environ. Microbiol..

[214] Ku J.W., Gan Y.H. (2019). Modulation of bacterial virulence and fitness by host glutathione. Curr. Opin. Microbiol..

[215] Masip L., Veeravalli K., Georgiou G. (2006). The many faces of glutathione in bacteria. Antioxid. Redox Signal..

[216] Landete J.M., De las Rivas B., Marcobal A., Muñoz R. (2008). Updated molecular knowledge about histamine biosynthesis by bacteria. Crit. Rev. Food Sci. Nutr..

[217] Dicks L.M.T. (2022). Gut bacteria and neurotransmitters. Microorganisms..

[218] Molenaar D., Bosscher J.S., ten Brink B., Driessen A.J., Konings W.N. (1993). Generation of a proton motive force by histidine decarboxylation and electrogenic histidine/histamine antiport in Lactobacillus buchneri. J. Bacteriol..

[219] Trip H., Mulder N.L., Lolkema J.S. (2012). Improved acid stress survival of *Lactococcus lactis* expressing the histidine decarboxylation pathway of *Streptococcus thermophilus* CHCC1524. J. Biol. Chem..

[220] Nugent S.G., Kumar D., Rampton D.S., Evans D.F. (2001). Intestinal luminal pH in inflammatory bowel disease: Possible determinants and implications for therapy with aminosalicylates and other drugs. Gut.

[221] Strandwitz P. (2018). Neurotransmitter modulation by the gut microbiota. Brain Res..

[222] Claus H., Decker H. (2006). Bacterial tyrosinases. Syst. Appl. Microbiol..

[223] Asano Y., Hiramoto T., Nishino R., Aiba Y., Kimura T., Yoshihara K., Koga Y., Sudo N. (2012). Critical role of gut microbiota in the production of biologically active, free catecholamines in the gut lumen of mice. Am. J. Physiol..

[224] Belay T., Sonnenfeld G. (2002). Differential effects of catecholamines on in vitro growth of pathogenic bacteria. Life Sci..

[225] Dichtl S., Demetz E., Haschka D., Tymoszuk P., Petzer V., Nairz M., Seifert M., Hoffmann A., Brigo N., Würzner R. (2019). Dopamine is a siderophore-like iron chelator that promotes *Salmonella enterica* serovar *Typhimurium virulence* in mice. mBio.

[226] Knecht L.D., O’Connor G., Mittal R., Liu X.Z., Daftarian P., Deo S.K., Daunert S. (2016). Serotonin activates bacterial quorum sensing and enhances the virulence of *Pseudomonas aeruginosa* in the host. EBioMedicine.

[227] Boyanova L. (2017). Stress hormone epinephrine (adrenaline) and norepinephrine (noradrenaline) effects on the anaerobic bacteria. Anaerobe.

[228] Lustri B.C., Sperandio V., Moreira C.G. (2017). Bacterial chat: Intestinal metabolites and signals in host-microbiota-pathogen interactions. Infect. Immun..

[229] O’Donnell P.M., Aviles H., Lyte M., Sonnenfeld G. (2006). Enhancement of in vitro growth of pathogenic bacteria by norepinephrine: Importance of inoculum density and role of transferrin. Appl. Environ. Microbiol..

[230] Kim J., Lee M.H., Kim M.S., Kim G.H., Yoon S.S. (2022). Probiotic properties and optimization of gamma-aminobutyric acid production by *Lactiplantibacillus plantarum* FBT215. J. Microbiol. Biotechnol..

[231] Barrett E., Ross R.P., O’Toole P.W., Fitzgerald G.F., Stanton C. (2012). gamma-aminobutyric acid production by culturable bacteria from the human intestine. J. Appl. Microbiol..

[232] Nomura M., Nakajima I., Fujita Y., Kobayashi M., Kimoto H., Suzuki I., Aso H. (1999). *Lactococcus lactis* contains only one glutamate decarboxylase gene. Microbiology.

[233] Otaru N., Ye K., Mujezinovic D., Berchtold L., Constancias F., Cornejo F.A., Krzystek A., de Wouters T., Braegger C., Lacroix C. (2021). GABA Production by human intestinal *Bacteroides* spp.: Prevalence, regulation, and role in acid stress tolerance. Front. Microbiol..

[234] Feehily C., Karatzas K.A. (2013). Role of glutamate metabolism in bacterial responses towards acid and other stresses. J. Appl. Microbiol..

[235] Prieto M.A., García J.L. (1997). Identification of the 4-hydroxyphenylacetate transport gene of Escherichia coli W: Construction of a highly sensitive cellular biosensor. FEBS Lett..

[236] YujiaLiu Shi C., Zhang G., Zhan H., Liu B., Li C., Wang L., Wang H., Wang J. (2021). Antimicrobial mechanism of 4-hydroxyphenylacetic acid on Listeria monocytogenes membrane and virulence. Biochem. Biophys. Res. Commun..

[237] Dai Z.L., Wu G., Zhu W.Y. (2011). Amino acid metabolism in intestinal bacteria: Links between gut ecology and host health. Front. Biosci..

[238] Endo A., Nakamura S., Konishi K., Nakagawa J., Tochio T. (2016). Variations in prebiotic oligosaccharide fermentation by intestinal lactic acid bacteria. Int. J. Food Sci. Nutr..

[239] Liong M.T., Shah N.P. (2005). Production of organic acids from fermentation of mannitol, fructooligosaccharide and inulin by a cholesterol removing *Lactobacillus acidophilus* strain. J. Appl. Microbiol..

[240] Goocher C.R., Woodside E.E., Kocholaty W. (1954). The influence of 2,4-dinitrophenol on the oxidation of acetate and succinate by Escherichia coli. J. Bacteriol..

[241] Lemma E., Hägerhäll C., Geisler V., Brandt U., von Jagow G., Kröger A. (1991). Reactivity of the Bacillus subtilis succinate dehydrogenase complex with quinones. Biochim. Biophys Acta.

[242] Adolph C., McNeil M.B., Cook G.M. (2022). Impaired succinate oxidation prevents growth and influences drug susceptibility in *Mycobacterium tuberculosis*. mBio..

[243] Hartman T., Weinrick B., Vilchèze C., Berney M., Tufariello J., Cook G.M., Jacobs W.R. (2014). Succinate dehydrogenase is the regulator of respiration in *Mycobacterium tuberculosis*. PLoS Pathog..

[244] Ferreyra J.A., Wu K.J., Hryckowian A.J., Bouley D.M., Weimer B.C., Sonnenburg J.L. (2014). Gut microbiota-produced succinate promotes C. difficile infection after antibiotic treatment or motility disturbance. Cell Host Microbe.

[245] Auria E., Deschamps J., Briandet R., Dupuy B. (2023). Extracellular succinate induces spatially organized biofilm formation in *Clostridioides difficile*. Biofilm.

[246] Hagihara M., Ariyoshi T., Kuroki Y., Eguchi S., Higashi S., Mori T., Nonogaki T., Iwasaki K., Yamashita M., Asai N. (2021). Clostridium butyricum enhances colonization resistance against *Clostridioides difficile* by metabolic and immune modulation. Sci. Rep..

[247] Shaulov Y., Shimokawa C., Trebicz-Geffen M., Nagaraja S., Methling K., Lalk M., Weiss-Cerem L., Lamm A.T., Hisaeda H., Ankri S. (2018). Escherichia coli mediated resistance of *Entamoeba histolytica* to oxidative stress is triggered by oxaloacetate. PLoS Pathog..

[248] Guillén N. (2023). Pathogenicity and virulence of *Entamoeba histolytica*, the agent of amoebiasis. Virulence.

[249] Kassem I.I., Candelero-Rueda R.A., Esseili K.A., Rajashekara G. (2017). Formate simultaneously reduces oxidase activity and enhances respiration in *Campylobacter jejuni*. Sci. Rep..

[250] Voskuhl L., Brusilova D., Brauer V.S., Meckenstock R.U. (2022). Inhibition of sulfate-reducing bacteria with formate. FEMS Microbiol. Ecol..

[251] Koestler B.J., Fisher C.R., Payne S.M. (2018). Formate promotes Shigella intercellular spread and virulence gene expression. mBio.

[252] Liu S.Q. (2003). Practical implications of lactate and pyruvate metabolism by lactic acid bacteria in food and beverage fermentations. Int. J. Food Microbiol..

[253] Sheridan P.O., Louis P., Tsompanidou E., Shaw S., Harmsen H.J., Duncan S.H., Flint H.J., Walker A.W. (2022). Distribution, organization and expression of genes concerned with anaerobic lactate utilization in human intestinal bacteria. Microb. Genom..

[254] Weghoff M.C., Bertsch J., Müller V. (2015). A novel mode of lactate metabolism in strictly anaerobic bacteria. Environ. Microbiol..

[255] Thauer R.K. (1988). Citric-acid cycle, 50 years on. Modifications and an alternative pathway in anaerobic bacteria. Eur. J. Biochem..

[256] Zampieri M., Hörl M., Hotz F., Müller N.F., Sauer U. (2019). Regulatory mechanisms underlying coordination of amino acid and glucose catabolism in Escherichia coli. Nat. Commun..

[257] Sarantinopoulos P., Makras L., Vaningelgem F., Kalantzopoulos G., De Vuyst L., Tsakalidou E. (2003). Growth and energy generation by Enterococcus faecium FAIR-E 198 during citrate metabolism. Int. J. Food Microbiol..

[258] Muñoz-Elías E.J., McKinney J.D. (2006). Carbon metabolism of intracellular bacteria. Cell Microbiol..

[259] Underhill S.A.M., Cabeen M.T. (2022). Redundancy in Citrate and cis-Aconitate Transport in *Pseudomonas aeruginosa*. J. Bacteriol..

[260] Mortera P., Pudlik A., Magni C., Alarcón S., Lolkema J.S. (2013). Ca^2+^-citrate uptake and metabolism in *Lactobacillus casei* ATCC 334. Appl. Environ. Microbiol..

[261] Pfenninger-Li X.D., Dimroth P. (1992). NADH formation by Na(+)-coupled reversed electron transfer in *Klebsiella pneumoniae*. Mol. Microbiol..

[262] Ramos A., Poolman B., Santos H., Lolkema J.S., Konings W.N. (1994). Uniport of anionic citrate and proton consumption in citrate metabolism generates a proton motive force in *Leuconostoc oenos*. J. Bacteriol..

[263] Pudlik A.M., Lolkema J.S. (2011). Citrate uptake in exchange with intermediates in the citrate metabolic pathway in *Lactococcus lactis* IL1403. J. Bacteriol..

[264] Sheldon J.R., Marolda C.L., Heinrichs D.E. (2014). TCA cycle activity in *Staphylococcus aureus* is essential for iron-regulated synthesis of staphyloferrin A, but not staphyloferrin B: The benefit of a second citrate synthase. Mol. Microbiol..

[265] Beaumont M., Portune K.J., Steuer N., Lan A., Cerrudo V., Audebert M., Dumont F., Mancano G., Khodorova N., Andriamihaja M. (2017). Quantity and source of dietary protein influence metabolite production by gut microbiota and rectal mucosa gene expression: A randomized, parallel, double-blind trial in overweight humans. Am. J. Clin. Nutr..

